# The effect of vitamin D supplementation on depression: a systematic review and dose–response meta-analysis of randomized controlled trials

**DOI:** 10.1017/S0033291724001697

**Published:** 2024-11

**Authors:** Shadi Ghaemi, Sheida Zeraattalab-Motlagh, Ahmad Jayedi, Sakineh Shab-Bidar

**Affiliations:** 1 Department of Community Nutrition, School of Nutritional Science and Dietetics, Tehran University of Medical Sciences (TUMS), Tehran, Iran; 2 Department of Health and Human Performance, University of Houston, Houston, Texas, USA; 3 Social Determinants of Health Research Center, Semnan University of Medical Sciences, Semnan, Iran; 4 Sports Medicine Research Center, Neuroscience Institute, Tehran University of Medical Sciences, Tehran, Iran

**Keywords:** anxiety, depression, dose–response, randomized controlled trials, Vitamin D_3_

## Abstract

The impact of vitamin D supplementation on depressive symptoms remains uncertain. This study aimed to investigate the dose-dependent effects of vitamin D supplementation on depressive and anxiety symptoms in adults. We systematically searched PubMed, Scopus, and Web of Science up to December 2022 to identify randomized controlled trials evaluating the effects of vitamin D_3_ supplementation on depression and anxiety symptoms in adults. Using a random-effects model, we calculated the standardized mean difference (SMD) for each 1000 IU/day vitamin D_3_ supplementation. The GRADE tool assessed the certainty of evidence. Our analysis included 31 trials with 24189 participants. Each 1000 IU/day vitamin D_3_ supplementation slightly reduced depressive symptoms in individuals with and without depression (SMD: −0.32, 95% CI −0.43 to −0.22; GEADE = moderate). The effect was more pronounced in those with depressive symptoms (SMD: −0.57, 95% CI −0.69 to −0.44; *n* = 15). The greatest reduction occurred at 8000 IU/day (SMD: −2.04, 95% CI −3.77 to −0.31). Trials with follow-up ⩽8 weeks (SMD: −0.45, 95% CI −0.70 to −0.20; *n* = 8) and 8 to ⩽24 weeks (SMD: −0.47, 95% CI −0.70 to −0.24; *n* = 15) showed stronger effects compared to those lasting 24 to ⩽52 weeks (SMD: −0.13, 95% CI −0.28 to 0.02; *n* = 5) or longer than 52 weeks (SMD: 0.14, 95% CI −0.16 to 0.44; *n* = 3) (*p* group difference <0.001). Vitamin D_3_ supplementation had no significant effects on anxiety symptoms. In summary, this study suggests that vitamin D_3_ supplementation may effectively reduce depressive symptoms in short term. Further high-quality trials are warranted for a conclusive assessment of its impact on anxiety.

## Introduction

Depression is the most common psychiatric disease worldwide, with an immense cognitive, social, and economic burden (Malhi & Mann, 2018). Currently, approximately 280 million people are affected by depression (GBD 2019 Diseases and Injuries Collaborators, 2020), making depression the leading cause of losing years of life due to disability (Friedrich, 2017; World Health, 2017). The World Health Organization ranked major depression as the third leading cause of disease worldwide and predicted that it will be the first public health concern by 2030 (World Health, 2008). Anxiety is also one of the most common mental health problems, with global estimates ranging from 3.8 to 25% across countries. Since 1990, there has been a 50% increase in the absolute number of patients with anxiety disorders (Z. Zhang et al., 2021). Additionally, research around the world has reported an increase in the prevalence of anxiety and depression symptoms during the COVID-19 outbreak (COVID-19 Mental Disorders Collaborators, 2021).

During the past decades, many prominent basic and clinical studies have been carried out to elucidate the underlying causes of depression (Saveanu & Nemeroff, 2012). A variety of factors including genetic predisposition, cognitive dysfunctions, stressful life events, interpersonal dysfunction, biological differences, and inherited traits are implicated in developing depression and anxiety (Altemus, Sarvaiya, & Neill Epperson, 2014; Hammen, 2018; Morssinkhof et al., 2020; Penner-Goeke & Binder, 2019). Studies in nutritional psychiatry have suggested some evidence of the role of dietary factors in developing depression and management of depressive symptoms (Marx et al., 2021; Pano et al., 2021). Furthermore, some studies investigated the effect of single micronutrient supplementation such as folate (Altaf, Gonzalez, Rubino, & Nemec, 2021; Jin et al., 2022), zinc (Yosaee et al., 2022), alpha-tocopherol (Lee et al., 2022), iron (Huang, Bilgrami, & Hare, 2022), and vitamin D (Parker, Brotchie, & Graham, 2017) on depression management. Of note, due to its possible role in the function of the brain, vitamin D has received much attention among micronutrients (Anglin, Samaan, Walter, & McDonald, 2013).

Vitamin D is a neuroprotective hormone, representing important roles in immune system regulation, promoting cell growth and differentiation, and anti-inflammatory pathways (Holick, 2007). It also regulates many essential genes involved in the brain function (Sultan et al., 2020). Several meta-analyses of epidemiological studies have suggested a positive relationship between vitamin D deficiency and risk of developing depression (Anglin et al., 2013; Ju, Lee, & Jeong, 2013).

Although some review studies have presented suggestions of a beneficial effect of vitamin D supplementation on depressive symptoms (Anglin et al., 2013; Cheng, Huang, & Huang, 2020; Mikola et al., 2023; Shaffer et al., 2014; Xie et al., 2022), none of these reviews have examined the potential dose-dependent effects of vitamin D supplementation on depressive symptoms to determine the optimum dose of intervention. Some of the available reviews, owing to the limited number of trials and methodological biases, were of low quality (Anglin et al., 2013; Cheng et al., 2020; Li et al., 2014; Shaffer et al., 2014). Considering these uncertainties, we aimed to fill this gap by conducting a systematic review and dose–response meta-analysis of randomized control trials (RCTs) to determine the optimum dose and shape of the effects of vitamin D supplementation on depression and anxiety symptoms in adults regardless of their health status.

## Methods

We conducted the meta-analysis per instructions outlined in the Preferred Reporting Items for Systematic Reviews and Meta-Analyses (PRISMA) 2020 statement for Systematic Reviews of Interventions (Page et al., 2021) and the Grading of Recommendations, Assessment, Development, and Evaluation (GRADE) Handbook (Schunemann, 2008). The review protocol was registered with PROSPERO (CRD42022309640).

### Data sources and searches

PubMed, Scopus, and Web of Science were systematically searched to find relevant literature up to 15 December 2022. Only English-language articles were included. Two investigators (SG and SZM) independently performed the systematic search and screened the titles and abstracts and then full-text articles to find eligible studies. Disagreements were resolved under the supervision of a third reviewer (SS-B). We also checked out the reference lists of published meta-analyses of RCTs of the effect of vitamin D supplementation. Further details about the search strategy in the databases are represented in online Supplementary Table 1.

### Study selection

Inclusion and exclusion criteria were defined according to the framework of PICOS (population, intervention/exposure, comparator, outcome, and study design). Published human intervention studies were considered eligible for inclusion in the present meta-analysis if they had the following criteria: (1) RCTs, with a minimum intervention period of 4 weeks, conducted in adults aged 18 years or older, regardless of medication use and their health status; (2) evaluated the effects of vitamin D supplementation alone, regardless of type (vitamin D_3_, vitamin D_2_), method of advice (orally, injection), and dosing regimen against a control group; (3) provided a dose of vitamin D supplementation in the intervention group; and (4) provided effect estimates in the form of mean difference and its 95% confidence interval (CI) for continuous outcomes and odds ratio (OR) with its 95% CI for binary outcome, or reported sufficient information to estimate those measures. Eligible control group included usual diet, no advice, no intervention, or placebo. Trials that compared two doses of vitamin D supplementation in intervention and control arms were eligible. Except trials that used vitamin D incombinaton with calcium, others that implemented a multicomponent intervention including vitamin D in combination with another supplement (for example, omega-3) were excluded from our review.

### Outcomes

Our primary outcomes were (1) the anxiety and depressive symptoms and (2) remission of depression. Moreover, our secondary outcomes included quality of life and its domains and total and serious adverse events.

### Data extraction

The full texts of potentially eligible trials were examined separately by two investigators (SG and SZM). From studies considered eligible, the same two reviewers independently extracted the following characteristics from each trial: author and year of publication; population location, study design, and duration, characteristics of the population (% female, mean age ± s.d., baseline body mass index, and health status), total sample size, intervention characteristics (dose of vitamin D supplementation in the intervention group), comparison group, vitamin D deficiency (yes/no/mixed), use of antidepressant medication (yes/no/mixed), dropout, baseline depression risk, outcome measures and main results for the outcomes included.

### Risk of bias assessment

Version 2.0 of the Cochrane tool was used to assess the risk of bias of the eligible RCTs (Higgins et al., 2011). Two authors (SG and SZM) independently performed risk of bias assessment. Disagreements were resolved by discussion with a third reviewer (SS-B).

### Data synthesis and analysis

We considered standardized mean difference (SMD) and its 95%CI for continuous outcomes (anxiety and depressive symptoms, comorbidity index, stress, overall quality of life, bodily image, dysphoria, food avoidance, healthy worry, relationship, sexual and social reaction) and OR and risk difference (RD) for binary outcomes (depression remission, adverse events, as well as serious adverse event) to report the results. For the analyses of continuous outcomes, we first computed the mean and s.d. of changes from baseline measures for each study arm in each trial. For the studies that did not report these changes, we estimated these values using the reported means and SDs of outcomes before and after the intervention per instructions in the Cochrane Handbook (Chandler, Cumpston, Li, Page, & Welch, 2019). We converted trial data to s.d. if they reported standard errors rather than s.d.. (Higgins & Green, 2008). If neither s.d. nor standard error were reported in the trials, we used the average s.d. obtained from other trials for the analyses (Furukawa, Barbui, Cipriani, Brambilla, & Watanabe, 2006). Second, we calculated SMD and its 95%CI for each 1000 IU/day vitamin D_3_ supplementation in each trial using the method introduced by Crippa and Orsini (Crippa & Orsini, 2016). Trial-specific results were pooled using a random-effect model (DerSimonian & Laird, 1986). The following items were needed for this method: the dose of vitamin D_3_ supplementation in the intervention and control arms, the mean and its related s.d. of change in outcomes in each study arm (treatment and control groups), and the total number of participants in each group. For trials that implemented vitamin D supplementation on a weekly or monthly basis (e.g. 30 000 IU per week or 50 000 IU per month), we converted the dosing regimen to IU per day. Finally, we conducted a dose–response meta-analysis to evaluate the shape of the dose-dependent effects of vitamin D_3_ supplementation on depressive and anxiety symptoms outcomes (Crippa & Orsini, 2016).

Then we performed pre-specified subgroup analyses based on the risk of bias (low/some concerns/high), intervention duration (⩽8, 9–24, 24–52, >52 weeks), sex (both/men/women), weight status (normal weight, obese/overweight, not reported), vitamin D deficiency (yes/no/mixed), health status, medication use (yes/no/mixed), and baseline risk of depression (low/medium/high). According to eight criteria determined by the Instrument to assess the Credibility of Effect Modification Analyses (ICEMAN), we examined the credibility of subgroup differences (Schandelmaier et al., 2020). We used meta-regression analyses to compute the *p*-values for subgroup differences. For publication bias, we applied Egger's test (Egger, Davey Smith, Schneider, & Minder, 1997) and Begg's test (Begg & Mazumdar, 1994) and inspected the funnel plots for asymmetry. The heterogeneity of effects among studies was quantified using the *I*^2^ statistic and *χ*2 test (P_heterogeneity_ > 0.10) (Higgins, Savovic, Page, Elbers, & Sterne, 2019).

For the analyses of binary outcomes (adverse event, depression remission), we calculated OR, RD, and their 95%CI using the number of participants and events in the intervention and control groups. Moreover, for depression remission judgment, every study defined specific criteria for recognition. For instance, in our review, only one study considered depression remission (Marsh, Penny, & Rothschild, 2017) and defined it as when participants had Montgomery-Åsberg Depression Rating Scale (MADRS) score was 12 or less. We used STATA software version 17.0 for our analyses. A two-tailed *p*-value less than 0.05 was regarded as statistically significant.

### Grading the evidence

The GRADE approach was applied to judge the certainty of the evidence (Guyatt et al., 2011). According to the GRADE, evidence obtained from RCTs starts at high certainty that can be downgraded or upgraded based on pre-defined criteria. Detailed criteria that were applied for use the GRADE tool are described in online Supplementary Table 2. In order to interpretation of the magnitude of effect sizes, the estimated SMDs were interpreted as a trivial effect (0.0–0.2), a small effect (0.2–0.6), a moderate effect (0.6–1.2), a large effect (1.2–2.0), a very large effect (2.0–4.0), and an extremely large effect (⩾4.0) (Hopkins, Marshall, Batterham, & Hanin, 2009; Varangot-Reille, Suso-Martí, Romero-Palau, Suárez-Pastor, & Cuenca-Martínez, 2022).

## Results

### Systematic search

The literature search yielded 2243 records, with 1 additional record identified through manual search. After excluding 607 duplicates and 1582 irrelevant records, 55 full texts were reviewed in detail for eligibility. After reviewing the full texts, 31 trials were eligible for inclusion in this dose–response meta-analysis (Alavi, Khademalhoseini, Vakili, & Assarian, 2019; Alghamdi et al., 2020; Bertone-Johnson et al., 2012; Choukri, Conner, Haszard, Harper, & Houghton, 2018; de Koning et al., 2019; Dean et al., 2011; Eid et al., 2019; Fazelian, Amani, Paknahad, Kheiri, & Khajehali, 2019; Frandsen, Pareek, Hansen, & Nielsen, 2014; Gaughran et al., 2021; Ghaderi et al., 2017, 2020; Hansen et al., 2019; Jorde & Kubiak, 2018; Jorde, Sneve, Figenschau, Svartberg, & Waterloo, 2008; Kaviani, Nikooyeh, Zand, Yaghmaei, & Neyestani, 2020; Khalighi Sikaroudi et al., 2020; Krivoy et al., 2017; Kusmiyati, Suryani, Herawati, & Firdausi, 2020; Marsh et al., 2017; Okereke et al., 2020; Omidian et al., 2019; Rolf et al., 2017; Sepehrmanesh et al., 2016; Sharifi, Vahedi, Nedjat, Mohamadkhani, & Hosseinzadeh Attar, 2019; Vellekkatt, Menon, Rajappa, & Sahoo, 2020; Vieth, Kimball, Hu, & Walfish, 2004; Wang et al., 2016; L. Zhang, Wang, Zhu, & Yang, 2018; Zheng et al., 2019; Zhu et al., 2020) (online Supplementary Figure 1). The list of studies excluded through full-text evaluation with reasons is reported in online Supplementary Table 3**.**

### Characteristics of original articles

Characteristics of 31 trials included in the present dose–response meta-analysis are presented in online Supplementary Table 4**.** The included trials comprised 24 189 participants, of which, 12 091 subjects were in the control arm and 12 098 subjects were in the intervention arm. Twenty-nine trials used vitamin D_3_ supplement as an intervention. In one of the RCTs, vitamin D supplementation was performed in combination with calcium (Bertone-Johnson et al., 2012). In another one of the RCTs, vitamin D supplementation was combined with omega-3 supplementation (Okereke et al., 2020). The intervention duration ranged from 4 to 256.8 weeks, of which, 8 trials lasted ⩽8 weeks (Alavi et al., 2019; Choukri et al., 2018; Dean et al., 2011; Kaviani et al., 2020; Krivoy et al., 2017; Kusmiyati et al., 2020; Sepehrmanesh et al., 2016; Zhang et al., 2018), fifteen trials lasted >8 to ⩽24 weeks (Alghamdi et al., 2020; Eid et al., 2019; Fazelian et al., 2019; Frandsen et al., 2014; Gaughran et al., 2021; Ghaderi et al., 2017, 2020; Hansen et al., 2019; Jorde & Kubiak, 2018; Khalighi Sikaroudi et al., 2020; Marsh et al., 2017; Omidian et al., 2019; Sharifi et al., 2019; Vellekkatt et al., 2020; Zhu et al., 2020), five trials lasted lasted >24 to ⩽52 weeks (de Koning et al., 2019; Jorde et al., 2008; Rolf et al., 2017; Vieth et al., 2004; Wang et al., 2016), and three trials lasted >52 weeks (Bertone-Johnson et al., 2012; Okereke et al., 2020; Zheng et al., 2019). Fifteen trials were conducted in patients with depression (Alavi et al., 2019; Alghamdi et al., 2020; Bertone-Johnson et al., 2012; Frandsen et al., 2014; Gaughran et al., 2021; Ghaderi et al., 2017, 2020; Hansen et al., 2019; Jorde et al., 2008; Kaviani et al., 2020; Omidian et al., 2019; Sepehrmanesh et al., 2016; Vellekkatt et al., 2020; Wang et al., 2016; Zhang et al., 2018), six trials were carried out on healthy subjects (Choukri et al., 2018; de Koning et al., 2019; Dean et al., 2011; Jorde & Kubiak, 2018; Kusmiyati et al., 2020; Okereke et al., 2020), two studies were carried out on subjects with anxiety (Eid et al., 2019; Fazelian et al., 2019), and other trials were carried out in patients with chronic diseases including schizophrenia (Krivoy et al., 2017), bipolar disorder (Marsh et al., 2017), multiple sclerosis (Rolf et al., 2017), ulcerative colitis (Sharifi et al., 2019), and irritable bowel syndrome (Khalighi Sikaroudi et al., 2020). Five of the 30 RCTs were carried out in individuals with normal weight (Choukri et al., 2018; Vellekkatt et al., 2020; Wang et al., 2016; Zheng et al., 2019; Zhu et al., 2020), 15 were carried out in individuals with overweight or obesity (body mass index ⩾25 kg/m^2^) (Alghamdi et al., 2020; de Koning et al., 2019; Fazelian et al., 2019; Gaughran et al., 2021; Ghaderi et al., 2017, 2020; Jorde & Kubiak, 2018; Jorde et al., 2008; Kaviani et al., 2020; Khalighi Sikaroudi et al., 2020; Krivoy et al., 2017; Omidian et al., 2019; Sepehrmanesh et al., 2016; Sharifi et al., 2019; Zheng et al., 2019), and the remaining 11 studies did not report the participants' weight status (Alavi et al., 2019; Bertone-Johnson et al., 2012; Dean et al., 2011; Eid et al., 2019; Frandsen et al., 2014; Hansen et al., 2019; Kusmiyati et al., 2020; Marsh et al., 2017; Okereke et al., 2020; Rolf et al., 2017; Vieth et al., 2004). Participants in the 17 trials had vitamin D deficiency (Alavi et al., 2019; de Koning et al., 2019; Eid et al., 2019; Fazelian et al., 2019; Ghaderi et al., 2017, 2020; Khalighi Sikaroudi et al., 2020; Krivoy et al., 2017; Marsh et al., 2017; Omidian et al., 2019; Rolf et al., 2017; Sepehrmanesh et al., 2016; Vellekkatt et al., 2020; Wang et al., 2016; Zhang et al., 2018; Zheng et al., 2019; Zhu et al., 2020), six trials were conducted in those without vitamin D deficiency (Choukri et al., 2018; Dean et al., 2011; Frandsen et al., 2014; Jorde & Kubiak, 2018; Rolf et al., 2017; Vieth et al., 2004), while the other eight trials were conducted in individuals with mixed vitamin D status (Alghamdi et al., 2020; Gaughran et al., 2021; Hansen et al., 2019; Jorde et al., 2008; Kaviani et al., 2020; Kusmiyati et al., 2020; Okereke et al., 2020; Sharifi et al., 2019). Five trials were rated as low risk of bias (Dean et al., 2011; Frandsen et al., 2014; Gaughran et al., 2021; Hansen et al., 2019; Vellekkatt et al., 2020), twelve trials were rated to have some concerns (Choukri et al., 2018; de Koning et al., 2019; Fazelian et al., 2019; Ghaderi et al., 2017, 2020; Jorde et al., 2008; Krivoy et al., 2017; Okereke et al., 2020; Omidian et al., 2019; Vieth et al., 2004; Wang et al., 2016; L. Zhang et al., 2018), and the other 14 trials were rated to have a high risk of bias (Alavi et al., 2019; Alghamdi et al., 2020; Bertone-Johnson et al., 2012; Eid et al., 2019; Jorde & Kubiak, 2018; Kaviani et al., 2020; Khalighi Sikaroudi et al., 2020; Kusmiyati et al., 2020; Marsh et al., 2017; Rolf et al., 2017; Sepehrmanesh et al., 2016; Sharifi et al., 2019; Zheng et al., 2019; Zhu et al., 2020) (online Supplementary Table 5**)**.

### Primary outcomes

A total of 31 trials with 12 098 participants in the intervention group and 12 091 participants in the control group provided data on the effect of vitamin D_3_ supplementation on the depressive symptoms (Alavi et al., 2019; Alghamdi et al., 2020; Bertone-Johnson et al., 2012; Choukri et al., 2018; de Koning et al., 2019; Dean et al., 2011; Eid et al., 2019; Fazelian et al., 2019; Frandsen et al., 2014; Gaughran et al., 2021; Ghaderi et al., 2017, 2020; Hansen et al., 2019; Jorde & Kubiak, 2018; Jorde et al., 2008; Kaviani et al., 2020; Khalighi Sikaroudi et al., 2020; Krivoy et al., 2017; Kusmiyati et al., 2020; Marsh et al., 2017; Okereke et al., 2020; Omidian et al., 2019; Rolf et al., 2017; Sepehrmanesh et al., 2016; Sharifi et al., 2019; Vellekkatt et al., 2020; Vieth et al., 2004; Wang et al., 2016; Zhang et al., 2018; Zheng et al., 2019; Zhu et al., 2020). Every 1000 IU/day vitamin D_3_ supplementation slightly reduced depressive symptoms (SMD: −0.32; 95% CI −0.43 to −0.22; *I*^2^ = 98%; P_heterogeneity_ < 0.001, GEADE = moderate) (online Supplementary Figure 2). Of note, vitamin D supplementation resulted in a larger improvement in depressive symptoms in those with a history of depression (SMD: −0.57, 95% CI −0.69 to −0.44; *n* = 15 trials) (×**Table 1**).

**Table 1.** Subgroup analyses of the effects of vitamin D_3_ supplement (each 1000 IU/d) on depressive symptoms

	*n*	Standardized mean difference (95% CI)	*I*^2^, P_heterogeneity_	P subgroup difference^a^
All trials	31	−0.32 (−0.43, −0.22)	98%, <0.001	–
Risk of bias				0.92
Low	5	−0.40 (−0.70, −0.09)	96%, <0.001	
Some concerns	12	−0.31 (−0.45, −0.19)	95%, <0.001	
High	14	−0.36 (−0.56, −0.15)	98%, <0.001	
Intervention duration				<0.001
⩽8 weeks	8	−0.45 (−0.70, −0.20)	95%, 0.41	
8 to ⩽24 weeks	15	−0.47 (−0.70, −0.24)	97%, <0.001	
24 to ⩽52 weeks	5	−0.13 (−0.28, 0.02)	97%, 0.01	
>52 weeks	3	0.14 (−0.16, 0.44)	98%, 0.54	
Sex				<0.001
Men	4	−0.21 (−0.43, 0.02)	39%, 0.22	
Women	1	−0.54 (−0.82, −0.47)	–	
Both	26	−0.34 (−0.45, −0.20)	98%, <0.001	
Weight status				0.01
Normal weight	5	−0.53 (−0.74, −0.33)	31%, 0.54	
Overweight/obese	15	−0.43 (−0.64, −0.21)	48%, 0.06	
Not reported	11	−0.11 (−0.29, 0.02)	91%, <0.001	
Vitamin D deficiency				<0.001
Yes	17	−0.26 (−0.42, −0.10)	98%, <0.001	
No	6	0.10 (−0.28, 0.08)	32%, 0.01	
Mixed	8	−0.91 (−1.29, −0.52)	96%, <0.001	
Health status				<0.001
Depressed	15	−0.57 (−0.69, −0.44)	96%, <0.001	
Healthy	6	−0.19 (−0.44, 0.06)	37%, 0.42	
Anxiety	2	−0.37 (−0.76, 0.01)	21%, 0.36	
Cognitive decline	1	−0.38 (−0.65, −0.12)	–	
Schizophrenia	1	0.55 (0.34, 0.86)	–	
Bipolar disorder	1	0.92 (0.66, 1.18)	–	
Multiple sclerosis	1	0.00 (−0.04, 0.04)	–	
Ulcerative colitis	1	−1.20 (−1.42, −0.98)	–	
Irritable bowel syndrome	1	−0.07 (−0.13, 0.00)	–	
Thyroid clinic outpatients	1	−0.35 (−0.50, −0.20)	–	
Knee osteoarthritis	1	0.39 (0.34, 0.45)	–	
Medication use				0.23
Yes	9	−0.54 (−0.85, −0.23)	66%, 0.01	
No	13	−0.28 (−0.40, −0.16)	97%, <0.001	
Mixed	3	−0.44 (−0.71, −0.18)	2%, 0.74	
Not reported	6	−0.51 (−0.79, −0.22)	55%, 0.04	
Baseline risk of depression				0.05
Low	5	−0.12 (−0.27, 0.02)	48%, 0.15	
Medium	5	−0.22 (−0.53, 0.10)	37%, 0.24	
High	21	−0.41 (−0.56, −0.25)	98%, <0.001	

a Obtained by meta-regression analysis.

Table 1 presents the subgroup analysis of the effects of vitamin D_3_ supplementation (each 1000 IU/day) on depressive symptoms. There were several potential effect modifications by intervention duration, sex, weight status, vitamin D status, and health status. However, the credibility of these subgroup differences was rated low (online Supplementary Table 6**)**. For instance, we found a stronger effect in trials with an intervention duration of ⩽8 weeks and 8 to ⩽24 weeks, and a nonsignificant effect in trials longer than 24 weeks (*p* group difference <0.001); however, only a few trials were available with follow-up longer than 52 weeks (Table 1). By using Egger's test (*p* = 0.23), Begg's test (*p* = 0.16), and an inspection of the funnel plot, we did not detect any evidence of publication bias **(**online Supplementary Figure 3**).**

**Figure 1.**
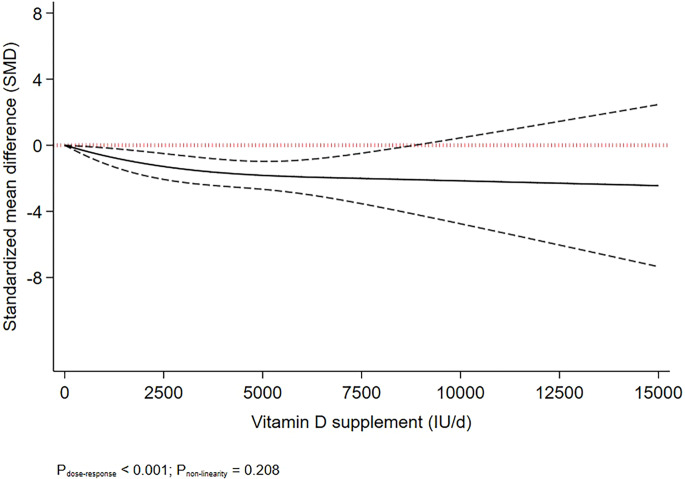
Dose-dependent effect of vitamin D supplement on depressive symptoms. Solid lines represent standardised mean difference and dashed lines represent 95% CI.

The effects of different doses of vitamin D_3_ on depressive symptoms are shown in Table 2 and Fig. 1. The analysis showed that depression symptoms decreased proportionally with increase in the dosage of vitamin D_3_ supplementation up to 8000 IU/day (SMD_8000 IU/d_: −2.04; 95% CI −3.77 to −0.31), with no further change in effect estimate at higher doses (P_dose−response_ < 0.001, P_nonlinearity_ = 0.208; *n* = 31, Fig. 1 and Table 2).

**Table 2.** The effects of vitamin D_3_ on depressive symptoms from the nonlinear dose–response meta-analysis (standardized mean difference and 95% confidence interval)

Vitamin D_3_ (IU/d)	0 (ref)	1000	3000	5000	6000	7000	8000	9000	12 000	15 000
Depressive symptoms	0	−0.63 (−1.10, −0.16)	−1.44 (−2.25, −0.63)	−1.82 (−2.66, −0.98)	−1.91 (−2.94, −0.89)	−1.98 (−3.32, −0.64)	−2.04 (−3.77, −0.31)	−2.09 (−4.25, 0.06)	−2.27 (−5.78, 1.24)	−2.44 (−7.35, 2.47)

One study reported the effect of vitamin D_3_ supplementation on the odds of depression remission (Marsh et al., 2017). Vitamin D_3_ supplementation had no significant effect on depression remission (online Supplementary Figure 4 and Table 3).

A total of seven trials with 332 individuals in the intervention group and 309 individuals in the control group provided data on the effect of vitamin D_3_ supplementation on the severity of anxiety (Choukri et al., 2018; Dean et al., 2011; Fazelian et al., 2019; Ghaderi et al., 2017, 2020; Khalighi Sikaroudi et al., 2020; Zhu et al., 2020). Our analysis showed that every 1000 IU/day of vitamin D_3_ did not significantly reduce the severity of anxiety (online Supplementary Figure 6 and Table 3).

### Secondary outcomes

The effect of vitamin D_3_ supplementation on secondary outcomes is demonstrated in online Supplementary Figure (7–20) and Table (3).<TE: Please check this is supplementary table citation or actual table ciation?. Opening bracket missing here.> Vitamin D_3_ supplementation had no effects on secondary outcomes including adverse events and different aspects of quality of life.

### Grading of the evidence

The GRADE evidence rating for primary and secondary outcomes is presented in online Supplementary Table 8. The certainty of the evidence was rated moderate for the effects of vitamin D_3_ supplementation on depressive symptoms due to a downgrade for inconsistency and an upgrade for the dose–response gradient. The certainty of evidence was rated very low to low for other outcomes (×**Table 3**). Of note, the effects of vitamin D_3_ supplementation on depressive symptoms surpassed the threshold set as large effect size (SMD: −1.2 to −2) in the nonlinear dose–response meta-analysis, suggesting that supplementation with vitamin D_3_ at a dose of 8000 IU/day could lead to a large improvement in depressive symptoms.

**Table 3.** The effect of vitamin D_3_ supplementation (per 1000 IU/day) on primary and secondary outcomes

Outcome (s)	Number of trials (participants)	Type of effect size	Effect size (95% CI)	GRADE certainty
Depressive symptoms	31 (24 189)	Standardized mean difference	−0.32 (−0.43, −0.22)	Moderate
Depression remission	1 (25)	Odds ratio	0.20 (0.03, 1.15)	Low
		Risk difference	−0.33 (−0.64, −0.03)	Low
Severity of anxiety	7 (641)	Standardized mean difference	−0.33 (−0.92, 0.25)	Low
Adverse events	8 (3672)	Odds ratio	1.20 (1.00, 1.45)	Very low
		Risk difference	0.01 (0.01, 0.02)	Low
Serious adverse events	2 (2115)	Odds ratio	1.00 (0.51, 1.97)	Low
		Risk difference	0.00 (−0.11, 0.11)	Low
Comorbidity index	1 (726)	Standardized mean difference	0.18 (−0.90, 1.26)	Low
Stress	1 (51)	Standardized mean difference	0.18 (−0.90, 1.26)	Low
Overall quality of life	2 (120)	Standardized mean difference	−0.24 (−0.63, 0.15)	Low
Bodily image	1 (74)	Standardized mean difference	−0.30 (−0.99, 0.39)	Low
Dysphoria	1 (74)	Standardized mean difference	−0.40 (−1.09, 0.29)	Low
Food avoidance	1 (74)	Standardized mean difference	0.02 (−0.41, 0.45)	Low
Healthy worry	1 (74)	Standardized mean difference	−0.16 (−0.63, 0.31)	Low
Relationship	1 (74)	Standardized mean difference	−0.13 (−0.50, 0.24)	Low
Sexual	1 (74)	Standardized mean difference	0.00 (−1.47, 1.47)	Low
Social reaction	1 (74)	Standardized mean difference	0.00 (−0.35, 0.35)	Low

## Discussion

To our knowledge, this is the first meta-analysis to investigate the dose-dependent effect of vitamin D supplementation on depression and anxiety symptoms in adults. Our results presented evidence of moderate certainty of the beneficial effects of vitamin D supplementation on depressive symptoms in adults. We did not find any significant effects of vitamin D supplementation on depression remission and anxiety symptoms. Vitamin D_3_ supplementation had no effects on quality of life.

Evidence from population-based epidemiological studies is consistent with the hypothesis that low serum vitamin D concentration is associated with a higher risk of depression (Anglin et al., 2013; Ju et al., 2013). Furthermore, our findings are consistent with existing evidence presented by meta-analysis of RCTs about the effects of vitamin D supplementation on depressive symptoms. A recent meta-analysis of 11 trials indicated that vitamin D_3_ supplementation improved the Beck Depression Inventory score in patients with psychiatric disorders (Jamilian et al., 2019). The results of another recent meta-analysis including 29 trials with 4504 participants indicated that the use of vitamin D_3_ may be beneficial to decrease the incidence of depression and improvement of depression symptoms (Xie et al., 2022). Moreover, another meta-analysis of four trials with 948 participants showed that vitamin D_3_ supplementation positively impacted depression rating in patients with major depression (Vellekkatt & Menon, 2019). However, there is some conflicting evidence, suggesting that oral administration of vitamin D_3_ did not have a significant effect on the reduction of post-intervention depression score (Lázaro Tomé et al., 2021).

Although the beneficial effects of vitamin D supplementation on depressive symptoms have been ascertained in previous research, the optimum dose of supplementation for reducing depressive symptoms has not been determined in previous studies. Our primary findings indicated that every 1000 IU/day of vitamin D_3_ supplementation significantly reduced depressive symptoms. We found that depressive symptoms decreased proportionally with the increase of the dosage of intervention up to 8000 IU/day. We found the greatest impact at 8000 IU/day, and that higher doses had no added values for treatment of depressive symptoms.

Our subgroup analyses indicated a stronger effect on depressive symptoms in women than in men. This is consistent with the literature, which suggests that the relationship between vitamin D_3_ deficiency and depression risk was stronger in women than in men (Harse et al., 2021; Landel, Annweiler, Millet, Morello, & Féron, 2016; Lerchbaum, 2014). There are physiological interactions between the effects of vitamin D and estrogen. Vitamin D deficiency may lead to lower estrogen levels, which can cause depression. This can make women more vulnerable to the effects of vitamin D deficiency (Harse et al., 2021; Landel et al., 2016; Lerchbaum, 2014). However, there was only one trial in women, and the credibility of subgroup difference was rated low. Therefore, the potential sex difference in the effect of vitamin D_3_ supplementation on depressive symptoms should be interpreted with caution.

Our subgroup analyses also highlighted a significant subgroup difference by intervention duration, where trials with an intervention duration of ⩽8 weeks and 8 to ⩽24 weeks indicated a stronger effect than those with 24 to ⩽52 weeks. Of note, an increasing effect was seen among trials with an intervention duration of >52 weeks. Regarding the length of the intervention, ‘8 weeks’ was identified as the window of time that may start the vitamin D response, whether in the prevention or therapy of depression (Xie et al., 2022). The decline in the efficacy of vitamin D supplementation in reducing depressive symptoms can be explained by attrition bias, lower compliance, and higher dropout in trials with longer intervention durations.

There are several possible physiological mechanisms to explain why vitamin D_3_ supplementation is effective in treating depression. These mechanisms include its impacts on cellular signaling, neurotropic and immunomodulatory processes, serotonin synthesis, expression of mitochondrial proteins, increasing the expression of vitamin D_3_ receptors in important brain regions, and decreasing levels of C-reactive protein, as an inflammatory marker linked to depression (Geng et al., 2019). Moreover, oxidative stress can be a contributing factor to depression. Vitamin D has important antioxidant properties and thus, may have therapeutic effects against depressive symptoms (Penckofer et al., 2022; Thurfah, Christine, Bagaskhara, Alfian, & Puspitasari, 2022; Vellekkatt & Menon, 2019). Vitamin D may also affect the serotonin system and maintain circadian rhythms, which are linked to depression (Casseb, Kaster, & Rodrigues, 2019; Ceolin, Mano, Hames, Antunes, & Moreira, 2021).

Additionally, the possible benefits of vitamin D on physical health in the long term were as follows: Vitamin D supplementation has been shown to have long-lasting benefits such as reducing the risk of falls and fractures, as demonstrated in the ViDA trial (Khaw et al., 2017). It has also been found to lower central blood pressure among vitamin D deficient participants (Sluyter et al., 2017) and lower the risk of overall mortality (Chowdhury et al., 2014).

Despite the aforementioned findings, we could not find a significant association between vitamin D_3_ supplementation and depression remission. Furthermore, we could not observe a significant relationship between vitamin D_3_ supplementation and the severity of anxiety. Nevertheless, some previous studies demonstrated that vitamin D supplementation was effective in alleviating anxiety symptoms (Borges-Vieira & Cardoso, 2023; Eid et al., 2019; Zaromytidou et al., 2022). These controversies may be attributed to the low number of trials available in this field, design of the studies, initial serum 25(OH)D concentrations of the participants, dose and type of vitamin D supplement (D_2_ v. D_3_), intervention duration, method of supplementation and age of the target group.

There were several strengths to our study. Our study updated earlier findings by including more recent RCTs in this field (Alavi et al., 2019; Alghamdi et al., 2020; de Koning et al., 2019; Eid et al., 2019; Fazelian et al., 2019; Gaughran et al., 2021; Ghaderi et al., 2020; Hansen et al., 2019; Kaviani et al., 2020; Kusmiyati et al., 2020; Omidian et al., 2019; Sharifi et al., 2019; Vellekkatt et al., 2020; Zheng et al., 2019; Zhu et al., 2020). Additionally, to the best of our knowledge, this is the first meta-analysis that investigated the dose-dependent effects of vitamin D_3_ supplementation on depression and anxiety symptoms in adults. We also applied the GRADE approach to rate the certainty of evidence. The Cochrane risk of bias tool (Version 2.0) was utilized to assess the methodological quality of included RCTs. Lastly, Pre-specified subgroup analyses were conducted based on various study characteristics, including risk of bias, intervention duration, sex, weight status, vitamin D deficiency, health status, medication use, and baseline risk of depression. This comprehensive approach enabled the exploration of potential effect modifiers.

However, there were also some limitations in the present meta-analysis. First, other factors may act as a moderator in the association between vitamin D supplementation and depressive symptoms. Although we did several subgroup analyses by participants and study characteristics, several unknown variables can affect the results. Second, we had limited evidence about the effects of vitamin D supplementation on depression remission and severity of anxiety. In addition, a subgroup analysis by intervention duration suggested that vitamin D supplementation was no longer effective in reducing depressive symptoms at follow-up longer than 52 weeks. Also, we included adults regardless of their depression, anxiety, and health status. Finally, despite efforts to identify all relevant studies, publication bias may have influenced the findings, as studies with statistically significant results are more likely to be published. Therefore, more trials are needed to test the long-term efficacy of vitamin D supplementation on depressive symptoms, as well as on depression remission and anxiety symptoms.

## Conclusion

Our dose–response meta-analysis of randomized trials demonstrated that vitamin D supplementation may result in a large improvement in depressive symptoms at follow-up shorter than 24 weeks. The greatest impact was seen at 8000 IU/day, and higher doses had no added value in reducing depressive symptoms. Vitamin D supplementation had no effects on depression remission and anxiety symptoms. More trials with high quality are needed to test the long-term efficacy of vitamin D supplementation, considering baseline vitamin D levels of individuals, on depressive symptoms, as well as on depression remission and anxiety symptoms.

## Supporting information

Ghaemi et al. supplementary material Ghaemi et al. supplementary material

## Data Availability

The data sets generated or analyzed during the current study are not publicly available but are available from the corresponding author at reasonable request.

## Supplementary material

The supplementary material for this article can be found at https://doi.org/10.1017/S0033291724001697.

## References

[ref1] Alavi, N. M., Khademalhoseini, S., Vakili, Z., & Assarian, F. (2019). Effect of vitamin D supplementation on depression in elderly patients: A randomized clinical trial. Clinical Nutrition, 38(5), 2065–2070. doi:10.1016/j.clnu.2018.09.011 30316534

[ref2] Alghamdi, S., Alsulami, N., Khoja, S., Alsufiani, H., Tayeb, H. O., & Tarazi, F. I. (2020). Vitamin D supplementation ameliorates severity of major depressive disorder. Journal of Molecular Neuroscience, 70(2), 230–235. doi:10.1007/s12031-019-01461-2 31836995

[ref3] Altaf, R., Gonzalez, I., Rubino, K., & Nemec, E. C. 2nd. (2021). Folate as adjunct therapy to SSRI/SNRI for major depressive disorder: Systematic review & meta-analysis. Complementary Therapies in Medicine, 61, 102770. doi:10.1016/j.ctim.2021.102770 34450256

[ref4] Altemus, M., Sarvaiya, N., & Neill Epperson, C. (2014). Sex differences in anxiety and depression clinical perspectives. Frontiers in Neuroendocrinology, 35(3), 320–330. doi:10.1016/j.yfrne.2014.05.004 24887405 PMC4890708

[ref5] Anglin, R. E., Samaan, Z., Walter, S. D., & McDonald, S. D. (2013). Vitamin D deficiency and depression in adults: Systematic review and meta-analysis. The British Journal of Psychiatry, 202, 100–107. doi:10.1192/bjp.bp.111.106666 23377209

[ref6] Begg, C. B., & Mazumdar, M. (1994). Operating characteristics of a rank correlation test for publication bias. Biometrics, 50(4), 1088–1101. 7786990

[ref7] Bertone-Johnson, E. R., Powers, S. I., Spangler, L., Larson, J., Michael, Y. L., Millen, A. E., … Manson, J. E. (2012). Vitamin D supplementation and depression in the women's health initiative calcium and vitamin D trial. American Journal of Epidemiology, 176(1), 1–13. doi:10.1093/aje/kwr482 22573431 PMC3385159

[ref8] Borges-Vieira, J. G., & Cardoso, C. K. S. (2023). Efficacy of B-vitamins and vitamin D therapy in improving depressive and anxiety disorders: A systematic review of randomized controlled trials. Nutritional Neuroscience, 26(3), 187–207. doi:10.1080/1028415x.2022.2031494 35156551

[ref9] Casseb, G. A., Kaster, M. P., & Rodrigues, A. L. S. (2019). Potential role of vitamin D for the management of depression and anxiety. CNS Drugs, 33(7), 619–637. 31093951 10.1007/s40263-019-00640-4

[ref10] Ceolin, G., Mano, G. P. R., Hames, N. S., Antunes, L. d. C., & Moreira, J. D. (2021). Vitamin D, depressive symptoms, and COVID-19 pandemic. Frontiers in Neuroscience, 15, 670879. 34054418 10.3389/fnins.2021.670879 PMC8155626

[ref11] Chandler, J., Cumpston, M., Li, T., Page, M., & Welch, V. (2019). Cochrane handbook for systematic reviews of interventions. Hoboken: Wiley.

[ref12] Cheng, Y. C., Huang, Y. C., & Huang, W. L. (2020). The effect of vitamin D supplement on negative emotions: A systematic review and meta-analysis. Depression and Anxiety, 37(6), 549–564. doi:10.1002/da.23025 32365423

[ref13] Choukri, M. A., Conner, T. S., Haszard, J. J., Harper, M. J., & Houghton, L. A. (2018). Effect of vitamin D supplementation on depressive symptoms and psychological wellbeing in healthy adult women: A double-blind randomised controlled clinical trial. Journal of Nutritional Science, 7, e23. doi:10.1017/jns.2018.14 30197783 PMC6123885

[ref14] Chowdhury, R., Kunutsor, S., Vitezova, A., Oliver-Williams, C., Chowdhury, S., Kiefte-de-Jong, J. C., … Hoshen, M. B. (2014). Vitamin D and risk of cause specific death: Systematic review and meta-analysis of observational cohort and randomised intervention studies. BMJ, 348, g1903. doi: 10.1136/bmj.g1903 24690623 PMC3972416

[ref200] COVID-19 Mental Disorders Collaborators. (2021). Global prevalence and burden of depressive and anxiety disorders in 204 countries and territories in 2020 due to the COVID-19 pandemic. The Lancet, 398(10312), 1700–1712. doi:10.1016/s0140-6736(21)02143-7 PMC8500697 34634250

[ref15] Crippa, A., & Orsini, N. (2016). Dose-response meta-analysis of differences in means. BMC Medical Research Methodology, 16, 91. doi:10.1186/s12874-016-0189-0 27485429 PMC4971698

[ref16] Dean, A. J., Bellgrove, M. A., Hall, T., Phan, W. M., Eyles, D. W., Kvaskoff, D., & McGrath, J. J. (2011). Effects of vitamin D supplementation on cognitive and emotional functioning in young adults--a randomised controlled trial. PLoS One, 6(11), e25966. doi:10.1371/journal.pone.0025966 22073146 PMC3208539

[ref17] de Koning, E. J., Lips, P., Penninx, B., Elders, P. J. M., Heijboer, A. C., den Heijer, M., … van Schoor, N. M. (2019). Vitamin D supplementation for the prevention of depression and poor physical function in older persons: The D-Vitaal study, a randomized clinical trial. The American Journal of Clinical Nutrition, 110(5), 1119–1130. doi:10.1093/ajcn/nqz141 31340012 PMC6821546

[ref18] DerSimonian, R., & Laird, N. (1986). Meta-analysis in clinical trials. Controlled Clinical Trials, 7(3), 177–188. doi:10.1016/0197-2456(86)90046-2 3802833

[ref19] Egger, M., Davey Smith, G., Schneider, M., & Minder, C. (1997). Bias in meta-analysis detected by a simple, graphical test. Bmj, 315(7109), 629–634. doi:10.1136/bmj.315.7109.629 9310563 PMC2127453

[ref20] Eid, A., Khoja, S., AlGhamdi, S., Alsufiani, H., Alzeben, F., Alhejaili, N., … Tarazi, F. I. (2019). Vitamin D supplementation ameliorates severity of generalized anxiety disorder (GAD). Metabolic Brain Disease, 34(6), 1781–1786. doi:10.1007/s11011-019-00486-1 31478182

[ref21] Fazelian, S., Amani, R., Paknahad, Z., Kheiri, S., & Khajehali, L. (2019). Effect of vitamin D supplement on mood status and inflammation in vitamin D deficient type 2 diabetic women with anxiety: A randomized clinical trial. International Journal of Preventive Medicine, 10, 17. doi:10.4103/ijpvm.IJPVM_174_18 30820304 PMC6390422

[ref22] Frandsen, T. B., Pareek, M., Hansen, J. P., & Nielsen, C. T. (2014). Vitamin D supplementation for treatment of seasonal affective symptoms in healthcare professionals: A double-blind randomised placebo-controlled trial. BMC Research Notes, 7, 528. doi:10.1186/1756-0500-7-528 25125215 PMC4141118

[ref23] Friedrich, M. J. J. J. (2017). Depression is the leading cause of disability around the world. JAMA, 317(15), 1517–1517. 10.1001/jama.2017.3826 28418490

[ref24] Furukawa, T. A., Barbui, C., Cipriani, A., Brambilla, P., & Watanabe, N. (2006). Imputing missing standard deviations in meta-analyses can provide accurate results. Journal of Clinical Epidemiology, 59(1), 7–10. doi:10.1016/j.jclinepi.2005.06.006 16360555

[ref25] Gaughran, F., Stringer, D., Wojewodka, G., Landau, S., Smith, S., Gardner-Sood, P., … McGrath, J. (2021). Effect of vitamin D supplementation on outcomes in people with early psychosis: The DFEND randomized clinical trial. JAMA Network Open, 4(12), e2140858. doi:10.1001/jamanetworkopen.2021.40858 34962559 PMC8715346

[ref201] GBD 2019 Diseases and Injuries Collaborators. (2020). Global burden of 369 diseases and injuries in 204 countries and territories, 1990-2019: A systematic analysis for the Global Burden of Disease Study 2019. The Lancet, 396(10258), 1204–1222. doi:10.1016/s0140-6736(20)30925-9. PMC7567026 33069326

[ref26] Geng, C., Shaikh, A. S., Han, W., Chen, D., Guo, Y., & Jiang, P. (2019). Vitamin D and depression: Mechanisms, determination and application. Asia Pacific Journal of Clinical Nutrition, 28(4), 689–694. 31826364 10.6133/apjcn.201912_28(4).0003

[ref27] Ghaderi, A., Banafshe, H. R., Motmaen, M., Rasouli-Azad, M., Bahmani, F., & Asemi, Z. (2017). Clinical trial of the effects of vitamin D supplementation on psychological symptoms and metabolic profiles in maintenance methadone treatment patients. Progress in Neuro-Psychopharmacology & Biological Psychiatry, 79(Pt B), 84–89. doi:10.1016/j.pnpbp.2017.06.016 28642082

[ref28] Ghaderi, A., Rasouli-Azad, M., Farhadi, M. H., Mirhosseini, N., Motmaen, M., Pishyareh, E., … Asemi, Z. (2020). Exploring the effects of vitamin D supplementation on cognitive functions and mental health status in subjects under methadone maintenance treatment. Journal of Addiction Medicine, 14(1), 18–25. doi:10.1097/adm.0000000000000550 31145174

[ref29] Guyatt, G. H., Oxman, A. D., Kunz, R., Brozek, J., Alonso-Coello, P., Rind, D., … Schünemann, H. J. (2011). GRADE guidelines 6. Rating the quality of evidence--imprecision. Journal of Clinical Epidemiology, 64(12), 1283–1293. doi:10.1016/j.jclinepi.2011.01.012 21839614

[ref32] Hammen, C. (2018). Risk factors for depression: An autobiographical review. Annual Review of Clinical Psychology, 14, 1–28. doi:10.1146/annurev-clinpsy-050817-084811 29328780

[ref33] Hansen, J. P., Pareek, M., Hvolby, A., Schmedes, A., Toft, T., Dahl, E., & Nielsen, C. T. (2019). Vitamin D3 supplementation and treatment outcomes in patients with depression (D3-vit-dep). BMC Research Notes, 12(1), 203. doi:10.1186/s13104-019-4218-z 30944021 PMC6446320

[ref34] Harse, J. D., Zhu, K., Bucks, R. S., Hunter, M., Lim, E. M., Cooke, B. R., … Murray, K. (2021). Investigating potential dose-response relationships between vitamin D status and cognitive performance: A cross-sectional analysis in middle- to older-aged adults in the Busselton healthy ageing study. International Journal of Environmental Research and Public Health, 19(1), 450. doi:10.3390/ijerph19010450 PMC8744852 35010710

[ref35] Higgins J. P., & Green, S. (2008). Selecting studies and collecting data. In J. P. T. Higgins and J. J. Deeks (Eds.), Cochrane handbook for systematic reviews of interventions: Cochrane book series (pp. 151–185).

[ref36] Higgins, J. P., Altman, D. G., Gøtzsche, P. C., Jüni, P., Moher, D., Oxman, A. D., … Sterne, J. A. (2011). The Cochrane Collaboration's tool for assessing risk of bias in randomised trials. Bmj, 343, d5928. doi:10.1136/bmj.d5928 22008217 PMC3196245

[ref37] Higgins, J. P., Savovic, J., Page, M. J., Elbers, R. G., & Sterne, J. A. (2019). Assessing risk of bias in a randomized trial. In J. P. T. Higgins, R. Churchill, J. Chandler, & M. S. Cumpston (Eds.),Cochrane handbook for systematic reviews of interventions (pp. 205–228).

[ref38] Holick, M. F. (2007). Vitamin D deficiency. The New England Journal of Medicine, 357(3), 266–281. doi:10.1056/NEJMra070553 17634462

[ref39] Hopkins, W. G., Marshall, S. W., Batterham, A. M., & Hanin, J. (2009). Progressive statistics for studies in sports medicine and exercise science. Medicine & Science in Sports & Exercise, 41(1), 3–13. doi:10.1249/MSS.0b013e31818cb278 19092709

[ref40] Huang, K. W., Bilgrami, N. L., & Hare, D. L. (2022). Iron deficiency in heart failure patients and benefits of iron replacement on clinical outcomes including comorbid depression. Heart, Lung and Circulation, 31(3), 313–326. doi:10.1016/j.hlc.2021.10.013 34810088

[ref41] Jamilian, H., Amirani, E., Milajerdi, A., Kolahdooz, F., Mirzaei, H., Zaroudi, M., … Asemi, Z. (2019). The effects of vitamin D supplementation on mental health, and biomarkers of inflammation and oxidative stress in patients with psychiatric disorders: A systematic review and meta-analysis of randomized controlled trials. Progress in Neuro-Psychopharmacology & Biological Psychiatry, 94, 109651. doi:10.1016/j.pnpbp.2019.109651 31095994

[ref42] Jin, X., Cheng, Z., Yu, X., Tao, Q., Huang, R., & Wang, S. (2022). Continuous supplementation of folic acid in pregnancy and the risk of perinatal depression-A meta-analysis. Journal of Affective Disorders, 302, 258–272. doi:10.1016/j.jad.2022.01.080 35066009

[ref43] Jorde, R., & Kubiak, J. (2018). No improvement in depressive symptoms by vitamin D supplementation: Results from a randomised controlled trial. Journal of Nutritional Science, 7, e30. doi:10.1017/jns.2018.19 30510695 PMC6262688

[ref44] Jorde, R., Sneve, M., Figenschau, Y., Svartberg, J., & Waterloo, K. (2008). Effects of vitamin D supplementation on symptoms of depression in overweight and obese subjects: Randomized double blind trial. Journal of Internal Medicine, 264(6), 599–609. doi:10.1111/j.1365-2796.2008.02008.x 18793245

[ref45] Ju, S. Y., Lee, Y. J., & Jeong, S. N. (2013). Serum 25-hydroxyvitamin D levels and the risk of depression: A systematic review and meta-analysis. Journal of Nutrition, Health & Aging, 17(5), 447–455. doi:10.1007/s12603-012-0418-023636546

[ref46] Kaviani, M., Nikooyeh, B., Zand, H., Yaghmaei, P., & Neyestani, T. R. (2020). Effects of vitamin D supplementation on depression and some involved neurotransmitters. Journal of Affective Disorders, 269, 28–35. doi:10.1016/j.jad.2020.03.029 32217340

[ref47] Khalighi Sikaroudi, M., Mokhtare, M., Shidfar, F., Janani, L., Faghihi Kashani, A., Masoodi, M., … Shidfar, S. (2020). Effects of vitamin D3 supplementation on clinical symptoms, quality of life, serum serotonin (5-hydroxytryptamine), 5-hydroxy-indole acetic acid, and ratio of 5-HIAA/5-HT in patients with diarrhea-predominant irritable bowel syndrome: A randomized clinical trial. EXCLI Journal, 19, 652–667. doi:10.17179/excli2020-2247 33013260 PMC7527498

[ref48] Khaw, K.-T., Stewart, A. W., Waayer, D., Lawes, C. M., Toop, L., Camargo, C. A., & Scragg, R. (2017). Effect of monthly high-dose vitamin D supplementation on falls and non-vertebral fractures: Secondary and post-hoc outcomes from the randomised, double-blind, placebo-controlled ViDA trial. The Lancet Diabetes & Endocrinology, 5(6), 438–447. 28461159 10.1016/S2213-8587(17)30103-1

[ref49] Krivoy, A., Onn, R., Vilner, Y., Hochman, E., Weizman, S., Paz, A., … Weizman, A. (2017). Vitamin D supplementation in chronic schizophrenia patients treated with clozapine: A randomized, double-blind, placebo-controlled clinical trial. EBioMedicine, 26, 138–145. doi:10.1016/j.ebiom.2017.11.027 29226809 PMC5832639

[ref50] Kusmiyati, Y., Suryani, E., Herawati, L., & Firdausi, A. (2020). Vitamin D and reduced academic stress of health students. Kesmas: National Public Health Journal, 15(3). doi:10.21109/kesmas.v15i3.3274

[ref51] Landel, V., Annweiler, C., Millet, P., Morello, M., & Féron, F. (2016). Vitamin D, cognition and Alzheimer's disease: The therapeutic benefit is in the D-tails. Journal of Alzheimer's Disease, 53(2), 419–444. doi:10.3233/jad-150943 PMC4969697 27176073

[ref52] Lázaro Tomé, A., Reig Cebriá, M. J., González-Teruel, A., Carbonell-Asíns, J. A., Cañete Nicolás, C., & Hernández-Viadel, M. (2021). Efficacy of vitamin D in the treatment of depression: A systematic review and meta-analysis. Actas Españolas de Psiquiatría, 49(1), 12–23. 33533015

[ref53] Lee, A., Tariq, A., Lau, G., Tok, N. W. K., Tam, W. W. S., & Ho, C. S. H. (2022). Vitamin E, alpha-tocopherol, and its effects on depression and anxiety: A systematic review and meta-analysis. Nutrients, 14(3), 656. doi:10.3390/nu14030656 PMC8840247 35277015

[ref54] Lerchbaum, E. (2014). Vitamin D and menopause--a narrative review. Maturitas, 79(1), 3–7. doi:10.1016/j.maturitas.2014.06.003 24993517

[ref55] Li, G., Mbuagbaw, L., Samaan, Z., Falavigna, M., Zhang, S., Adachi, J. D., … Thabane, L. (2014). Efficacy of vitamin D supplementation in depression in adults: A systematic review. The Journal of Clinical Endocrinology & Metabolism, 99(3), 757–767. doi:10.1210/jc.2013-3450 24423304 PMC5112012

[ref56] Malhi, G. S., & Mann, J. J. (2018). Depression. The Lancet, 392(10161), 2299–2312. doi:10.1016/s0140-6736(18)31948-2 30396512

[ref57] Marsh, W. K., Penny, J. L., & Rothschild, A. J. (2017). Vitamin D supplementation in bipolar depression: A double blind placebo controlled trial. Journal of Psychiatric Research, 95, 48–53. doi:10.1016/j.jpsychires.2017.07.021 28777983

[ref58] Marx, W., Lane, M., Hockey, M., Aslam, H., Berk, M., Walder, K., … Jacka, F. N. (2021). Diet and depression: Exploring the biological mechanisms of action. Molecular Psychiatry, 26(1), 134–150. doi:10.1038/s41380-020-00925-x 33144709

[ref59] Mikola, T., Marx, W., Lane, M. M., Hockey, M., Loughman, A., Rajapolvi, S., … Ruusunen, A. (2023). The effect of vitamin D supplementation on depressive symptoms in adults: A systematic review and meta-analysis of randomized controlled trials. Critical Reviews in Food Science and Nutrition, *63*(33), 11784–11801. doi:10.1080/10408398.2022.2096560 35816192

[ref60] Morssinkhof, M. W. L., van Wylick, D. W., Priester-Vink, S., van der Werf, Y. D., den Heijer, M., van den Heuvel, O. A., & Broekman, B. F. P. (2020). Associations between sex hormones, sleep problems and depression: A systematic review. Neuroscience and Biobehavioral Reviews, 118, 669–680. doi:10.1016/j.neubiorev.2020.08.006 32882313

[ref61] Okereke, O. I., Reynolds, C. F. 3rd, Mischoulon, D., Chang, G., Vyas, C. M., Cook, N. R., … Manson, J. E. (2020). Effect of long-term vitamin D3 supplementation vs placebo on risk of depression or clinically relevant depressive symptoms and on change in mood scores: A randomized clinical trial. JAMA, 324(5), 471–480. doi:10.1001/jama.2020.10224 32749491 PMC7403921

[ref62] Omidian, M., Mahmoudi, M., Abshirini, M., Eshraghian, M. R., Javanbakht, M. H., Zarei, M., … Djalali, M. (2019). Effects of vitamin D supplementation on depressive symptoms in type 2 diabetes mellitus patients: Randomized placebo-controlled double-blind clinical trial. Diabetes & Metabolic Syndrome, 13(4), 2375–2380. doi:10.1016/j.dsx.2019.06.011 31405646

[ref63] Page, M. J., McKenzie, J. E., Bossuyt, P. M., Boutron, I., Hoffmann, T. C., Mulrow, C. D., … Brennan, S. E. (2021). The PRISMA 2020 statement: An updated guideline for reporting systematic reviews. International Journal of Surgery, 88, 105906. 33789826 10.1016/j.ijsu.2021.105906

[ref64] Pano, O., Martínez-Lapiscina, E. H., Sayón-Orea, C., Martinez-Gonzalez, M. A., Martinez, J. A., & Sanchez-Villegas, A. (2021). Healthy diet, depression and quality of life: A narrative review of biological mechanisms and primary prevention opportunities. World Journal of Psychiatry, 11(11), 997–1016. doi:10.5498/wjp.v11.i11.997 34888169 PMC8613751

[ref65] Parker, G. B., Brotchie, H., & Graham, R. K. (2017). Vitamin D and depression. Journal of Affective Disorders, 208, 56–61. doi:10.1016/j.jad.2016.08.082 27750060

[ref66] Penckofer, S., Ridosh, M., Adams, W., Grzesiak, M., Woo, J., Byrn, M., … Halaris, A. (2022). Vitamin D supplementation for the treatment of depressive symptoms in women with type 2 diabetes: A randomized clinical trial. Journal of Diabetes Research, 2022, 4090807. doi:10.1155/2022/4090807 35280228 PMC8913152

[ref67] Penner-Goeke, S., & Binder, E. B. (2019). Epigenetics and depression. Dialogues in Clinical Neuroscience, 21(4), 397–405. doi:10.31887/DCNS.2019.21.4/ebinder 31949407 PMC6952745

[ref68] Rolf, L., Muris, A. H., Bol, Y., Damoiseaux, J., Smolders, J., & Hupperts, R. (2017). Vitamin D(3) supplementation in multiple sclerosis: Symptoms and biomarkers of depression. Journal of the Neurological Sciences, 378, 30–35. doi:10.1016/j.jns.2017.04.017 28566173

[ref69] Saveanu, R. V., & Nemeroff, C. B. (2012). Etiology of depression: Genetic and environmental factors. Psychiatric Clinics of North America, 35(1), 51–71. doi:10.1016/j.psc.2011.12.001 22370490

[ref70] Schandelmaier, S., Briel, M., Varadhan, R., Schmid, C. H., Devasenapathy, N., Hayward, R. A., … Guyatt, G. H. (2020). Development of the Instrument to assess the Credibility of Effect Modification Analyses (ICEMAN) in randomized controlled trials and meta-analyses. Canadian Medical Association Journal, 192(32), E901–e906. doi:10.1503/cmaj.200077 32778601 PMC7829020

[ref71] Schunemann, H. (2008). GRADE handbook for grading quality of evidence and strength of recommendation. *BMJ*, *336*(7653):1106–1110. doi:10.1136/bmj.39500.677199.AE PMC2386626 18483053

[ref72] Sepehrmanesh, Z., Kolahdooz, F., Abedi, F., Mazroii, N., Assarian, A., Asemi, Z., & Esmaillzadeh, A. (2016). Vitamin D supplementation affects the Beck depression inventory, insulin resistance, and biomarkers of oxidative stress in patients with major depressive disorder: A randomized, controlled clinical trial. The Journal of Nutrition, 146(2), 243–248. doi:10.3945/jn.115.218883 26609167

[ref73] Shaffer, J. A., Edmondson, D., Wasson, L. T., Falzon, L., Homma, K., Ezeokoli, N., … Davidson, K. W. (2014). Vitamin D supplementation for depressive symptoms: A systematic review and meta-analysis of randomized controlled trials. Psychosomatic Medicine, 76(3), 190–196. doi:10.1097/psy.0000000000000044 24632894 PMC4008710

[ref74] Sharifi, A., Vahedi, H., Nedjat, S., Mohamadkhani, A., & Hosseinzadeh Attar, M. J. (2019). Vitamin D decreases Beck depression inventory score in patients with mild to moderate ulcerative colitis: A double-blind randomized placebo-controlled trial. Journal of Dietary Supplements, 16(5), 541–549. doi:10.1080/19390211.2018.1472168 29958055

[ref75] Sluyter, J. D., Camargo C. A. Jr., Stewart, A. W., Waayer, D., Lawes, C. M., Toop, L., … Wassertheurer, S. (2017). Effect of monthly, high-dose, long-term vitamin D supplementation on central blood pressure parameters: A randomized controlled trial substudy. Journal of the American Heart Association, 6(10), e006802. 29066444 10.1161/JAHA.117.006802 PMC5721873

[ref76] Sultan, S., Taimuri, U., Basnan, S. A., Ai-Orabi, W. K., Awadallah, A., Almowald, F., & Hazazi, A. (2020). Low Vitamin D and its association with cognitive impairment and dementia. Journal of Aging Research, 2020, 6097820. doi:10.1155/2020/6097820 32399297 PMC7210535

[ref77] Thurfah, J. N., Christine, Bagaskhara, P. P., Alfian, S. D., & Puspitasari, I. M. (2022). Dietary supplementations and depression. Journal of Multidisciplinary Healthcare, 15, 1121–1141. doi:10.2147/jmdh.S360029 35607362 PMC9123934

[ref78] Varangot-Reille, C., Suso-Martí, L., Romero-Palau, M., Suárez-Pastor, P., & Cuenca-Martínez, F. (2022). Effects of different therapeutic exercise modalities on migraine or tension-type headache: A systematic review and meta-analysis with a replicability analysis. The Journal of Pain, 23(7), 1099–1122. doi:10.1016/j.jpain.2021.12.003 34929374

[ref79] Vellekkatt, F., & Menon, V. (2019). Efficacy of vitamin D supplementation in major depression: A meta-analysis of randomized controlled trials. Journal of Postgraduate Medicine, 65(2), 74–80. doi:10.4103/jpgm.JPGM_571_17 29943744 PMC6515787

[ref80] Vellekkatt, F., Menon, V., Rajappa, M., & Sahoo, J. (2020). Effect of adjunctive single dose parenteral Vitamin D supplementation in major depressive disorder with concurrent vitamin D deficiency: A double-blind randomized placebo-controlled trial. Journal of Psychiatric Research, 129, 250–256. doi:10.1016/j.jpsychires.2020.07.037 32823218

[ref81] Vieth, R., Kimball, S., Hu, A., & Walfish, P. G. (2004). Randomized comparison of the effects of the vitamin D3 adequate intake versus 100 mcg (4000 IU) per day on biochemical responses and the wellbeing of patients. Nutrition Journal, 3, 8. doi:10.1186/1475-2891-3-8 15260882 PMC506781

[ref82] Wang, Y., Liu, Y., Lian, Y., Li, N., Liu, H., & Li, G. (2016). Efficacy of high-dose supplementation with oral vitamin D3 on depressive symptoms in dialysis patients with vitamin D3 insufficiency: A prospective, randomized, double-blind study. Journal of Clinical Psychopharmacology, 36(3), 229–235. doi:10.1097/jcp.0000000000000486 27022679

[ref83] World Health, O. (2008). The global burden of disease : 2004 update. Geneva: World Health Organization.

[ref84] World Health, O. (2017). Depression and other common mental disorders: global health estimates. Retrieved from Geneva: https://apps.who.int/iris/handle/10665/254610

[ref85] Xie, F., Huang, T., Lou, D., Fu, R., Ni, C., Hong, J., & Ruan, L. (2022). Effect of vitamin D supplementation on the incidence and prognosis of depression: An updated meta-analysis based on randomized controlled trials. Frontiers in Public Health, 10, 903547. doi:10.3389/fpubh.2022.903547 35979473 PMC9376678

[ref86] Yosaee, S., Clark, C. C. T., Keshtkaran, Z., Ashourpour, M., Keshani, P., & Soltani, S. (2022). Zinc in depression: From development to treatment: A comparative/ dose response meta-analysis of observational studies and randomized controlled trials. General Hospital Psychiatry, 74, 110–117. doi:10.1016/j.genhosppsych.2020.08.001 32829928

[ref87] Zaromytidou, E., Koufakis, T., Dimakopoulos, G., Drivakou, D., Konstantinidou, S., Rakitzi, P., … Kotsa, K. (2022). Vitamin D alleviates anxiety and depression in elderly people with prediabetes: A randomized controlled study. Metabolites, 12(10), 884. doi:10.3390/metabo12100884 PMC9611739 36295786

[ref88] Zhang, L., Wang, S., Zhu, Y., & Yang, T. (2018). Vitamin D3 as adjunctive therapy in the treatment of depression in tuberculosis patients: A short-term pilot randomized double-blind controlled study. Neuropsychiatric Disease and Treatment, 14, 3103–3109. doi:10.2147/ndt.S183039 30532541 PMC6241718

[ref89] Zhang, Z., Wu, Y., Zhong, C., Zhou, X., Liu, C., Li, Q., … Yang, N. (2021). Association between dietary inflammatory index and gestational diabetes mellitus risk in a prospective birth cohort study. Nutrition (Burbank, Los Angeles County, Calif.), 87-88, 111193. doi:10.1016/j.nut.2021.111193 33774421

[ref90] Zheng, S., Tu, L., Cicuttini, F., Han, W., Zhu, Z., Antony, B., … Ding, C. (2019). Effect of vitamin D supplementation on depressive symptoms in patients with knee osteoarthritis. Journal of the American Medical Directors Association, 20(12), 1634–1640.e1631. doi:10.1016/j.jamda.2018.09.006 30401608

[ref91] Zhu, C., Zhang, Y., Wang, T., Lin, Y., Yu, J., Xia, Q., … Zhu, D. M. (2020). Vitamin D supplementation improves anxiety but not depression symptoms in patients with vitamin D deficiency. Brain and Behavior, 10(11), e01760. doi:10.1002/brb3.1760 32945627 PMC7667301

