# Supplementary material for: The effect of vitamin D supplementation on depression: a systematic review and dose–response meta-analysis of randomized controlled trials
Source: Psychol Med. 2024 Nov 18;54(15):3999–4008. doi: 10.1017/S0033291724001697 (PMC11650176; doi:10.1017/S0033291724001697)
Supplement: Ghaemi et al. supplementary material [file S0033291724001697sup001.doc]

**The effect of vitamin D3 supplementation on depression: a systematic review and dose-response meta-analysis of randomized controlled trials**

Supplementary Tables 1-7 and Supplementary Figures 1-20

**Supplementary Table 1.** Search strategy (PubMed) to find potential eligible trials for inclusion in dose-response meta-analysis of the effect of vitamin D3 supplementation on depression (December 2022).

| 1. Vitamin D[tiab] OR "vitamin D2"[tiab] OR "vitamin D3"[tiab] OR calcitriol[tiab] OR cholecalciferol[tiab] OR "1-alpha hydroxyvitamin D3"[tiab] OR "1,25-dihydroxyvitamin D3"[tiab] OR "1,25 dihydroxycholecalciferol"[tiab] OR "1,25-dihydroxyvitamin D"[tiab] OR "25 hydroxyvitamin"[tiab] OR 25-hydroxy-vitamin[tiab] OR "25 hydroxycholecalciferol"[tiab] OR "25-hydroxyvitamin D"[tiab] OR "vitamin D supplementation"[tiab] OR ergocalciferol[tiab] OR calcifediol[tiab] OR alfa-calcidol[tiab] OR calcidiol[tiab] OR calciferol[tiab] OR ergocalciferol[tiab] OR 25-OH-D[tiab] OR Hydroxycholecalciferol[tiab] OR “25(OH)D”[tiab] OR “25(OH) vitamin D”[tiab] OR "Vitamin D"[Mesh] OR "Cholecalciferol"[Mesh] OR "Calcitriol"[Mesh] OR "1,25-dihydroxyvitamin D" [Supplementary Concept] OR "Calcifediol"[Mesh] OR "25-Hydroxyvitamin D 2"[Mesh] OR "Ergocalciferols"[Mesh] |
| --- |
| 2. Depression[tiab] OR “mental health”[tiab] OR “brain volume”[tiab] OR “brain structure”[tiab] OR “white matter”[tiab] OR depress[tiab] OR mood[tiab] OR anxiety[tiab] OR anxious[tiab] OR “Depressive Disorder”[tiab] OR “Depressive Neuroses”[tiab] OR “Endogenous Depression”[tiab] OR “Depressive Syndrome”[tiab] OR "Psychotic Affective Disorders"[tiab] OR “Neurotic Depression”[tiab] OR Melancholia[tiab] OR “Unipolar Depression”[tiab] OR “bipolar depression”[tiab] OR “affective disorders”[tiab] OR “Major Depressive Disorder”[tiab] OR “Involutional Psychoses”[tiab] OR “Involutional Psychosis”[tiab] OR “Involutional Depression”[tiab] OR “depressive symptom”[tiab] OR dysthymia[tiab] OR Depression [Mesh] OR "Mental Health"[Mesh] OR "Anxiety"[Mesh] OR "Depressive Disorder"[Mesh] OR "Bipolar Disorder"[Mesh] OR "Affective Disorders, Psychotic"[Mesh] |
| 3. intervention[tiab] OR RCT[tiab] OR "controlled trial"[tiab] OR randomized[tiab] OR random[tiab] OR Randomly[tiab] OR Placebo[tiab] OR Assignment[tiab] OR "clinical trial"[tiab] OR trial[tiab] OR randomised[tiab] OR "Methods"[Mesh] OR "Randomized Controlled Trial"[Publication Type] OR "Controlled Clinical Trial"[Publication Type] OR "Placebos"[Mesh] OR "Placebo Effect"[Mesh] OR "Clinical Trial"[Publication Type] OR "Clinical Trials as Topic"[Mesh] |
| 14. 1 AND 2 AND 3 |

**Supplementary Table 2.** Instructions to rate the certainty of evidence using the GRADE approach.

| GRADE domain (Guyatt et al., 2008) | Relevant content |
| --- | --- |
| **Criteria for downgrading** | |
| Risk of bias (also known as study limitation) (Guyatt, Oxman, Vist, et al., 2011) | -Rated down for imprecision if most studies were at high risk of bias |
| Inconsistency (Guyatt, Oxman, Kunz, Woodcock, Brozek, Helfand, Alonso-Coello, Glasziou, et al., 2011) | -Substantial between-study heterogeneity, I^2^ ≥ 50% and P_heterogeneity_< 0.10 which remained unexplained in priori subgroup and sensitivity analyses |
| Indirectness (Guyatt, Oxman, Kunz, Woodcock, Brozek, Helfand, Alonso-Coello, Falck-Ytter, et al., 2011) | -Presence of population, intervention or comparator factors that limit the generalizability of the results |
| Imprecision (Guyatt, Oxman, Kunz, Brozek, et al., 2011) | -The 95% CI for the mean difference is wide or the point estimate and its corresponding 95%CI do not surpass the MCID  -We did not rate down imprecision where the point estimate and its 95%CI for the linear and/or non-linear dose-response meta-analyses surpassed MCID thresholds. Where the point estimates surpassed MCID, we rated down if the 95%CI overlapped that threshold. We did not rate down for imprecision if point estimate and its 95%CI surpassed MCID thresholds at any specific dose of intervention in the non-linear dose-response meta-analysis. |
| Publication bias (Guyatt, Oxman, Montori, et al., 2011) | -Compelling evidence of publication bias |
| **Criteria for upgrading** | |
| Large effect size | -Explicit description of the magnitude of effect considered as large |
| Presence of a dose-response gradient | -Whether the studies provide evidence of a dose-response gradient between intervention or exposure and outcome |
| **Overall rating** | |
| Rating certainty of evidence | -High  - Moderate  -Low |

CI, confidence interval; MCID, minimal clinically important difference.

**Supplementary Table 3.** Excluded studies from the search of databases (n=24).

| **NO** | **References** | **Reason for exclusion** |
| --- | --- | --- |
| **1** | Abiri et al. Randomized study of the effects of vitamin D and/or magnesium supplementation on mood, serum levels of BDNF, inflammation, and SIRT1 in obese women with mild to moderate depressive symptoms. | Multivitamin supplementation |
| **2** | Byrn et al. Vitamin D Supplementation and Cognition in People with Type 2 Diabetes: A Randomized Control Trial. | Not sufficient information |
| **3** | Cheema & Chaudhry. Quality-of-life indicators and falls due to vitamin D deficiency. | Not randomized controlled trial |
| **4** | Dean et al. Effects of vitamin D supplementation on cognitive and emotional functioning in young adults--a randomised controlled trial. | Duplicate |
| **5** | De koning et al. Vitamin D supplementation for the prevention of depression and poor physical function in older persons: the D-Vitaal study, a randomized clinical trial. | Duplicate |
| **6** | Khalighi Sikaroudi et al. Effects of vitamin D3 supplementation on clinical symptoms, quality of life, serum serotonin (5-hydroxytryptamine), 5-hydroxy-indole acetic acid, and ratio of 5-HIAA/5-HT in patients with diarrhea-predominant irritable bowel syndrome: A randomized clinical trial. | Duplicate |
| **7** | Kaplan et al. A randomised trial of nutrient supplements to minimise psychological stress after a natural disaster. | Multivitamin supplementation |
| **8** | Lee et al. Effects of Vitamin D on Depression, Cognitive Function, and Physical Function in Elderly Individuals Living Alone. | Not randomized controlled trial |
| **9** | Lansdowne & Provost. Vitamin D3 enhances mood in healthy subjects during winter. | Not sufficient information |
| **10** | Korokosz et al. Personality Traits and Vitamin D3 Supplementation Affect Mood State 12 h Before 100 km Ultramarathon Run. | Under 4 weeks |
| **11** | Mehrabani et al. The Effect of Vitamin D3 on Depression in Iranian Women. | Not sufficient information |
| **12** | Okereke et al. Effect of Long-term Vitamin D3 Supplementation vs Placebo on Risk of Depression or Clinically Relevant Depressive Symptoms and on Change in Mood Scores. | Not interested intervention |
| **13** | Penckofer et al. Vitamin D Supplementation Improves Mood in Women with Type 2 Diabetes. | Not sufficient information |
| **14** | Sanders et al. Annual high-dose vitamin D3 and mental well-being: randomised controlled trial. | Not sufficient information |
| **15** | Samadi et al. Cardiac Rehabilitation Program and Vitamin D Supplement on NO, ET1, VEGF, Anxiety, and Depression Levels in Cardiac Patients after Coronary Artery Bypass Grafting. | Not sufficient information |
| **16** | Torrisi et al. The role of rehabilitation and vitamin D supplementation on motor and psychological outcomes in poststroke patients. | Not sufficient information |
| **17** | Veleva et al. The Effect of Ultraviolet B Irradiation Compared with Oral Vitamin D Supplementation on the Well-being of Nursing Home Residents with Dementia: A Randomized Controlled Trial. | Not sufficient information |
| **18** | Vellekkatt et al. Effect of adjunctive single dose parenteral Vitamin D supplementation in major depressive disorder with concurrent vitamin D deficiency: A double-blind randomized placebo-controlled trial | Duplicate |
| **19** | Westra et al. Effect of vitamin D supplementation on health status in non-vitamin D deficient people with type 2 diabetes mellitus. | Not interested outcome |
| **20** | Yalamanchili & Gallagher. Dose ranging effects of Vitamin D3 on the Geriatric Depression Score: A Clinical Trial. | Not sufficient information |
| **21** | Wang et al. Effects of vitamin C and vitamin D administration on mood and distress in acutely hospitalized patients. | Multivitamin supplementation |
| **22** | Yalamanchili & Gallagher. Treatment with hormone therapy and calcitriol did not affect depression in older postmenopausal women: no interaction with estrogen and vitamin D receptor genotype polymorphisms | Not interested intervention |
| **23** | Yosaee et al. Effects of zinc, vitamin D, and their co-supplementation on mood, serum cortisol, and brain-derived neurotrophic factor in patients with obesity and mild to moderate depressive symptoms: A phase II, 12-wk, 2 × 2 factorial design, double-blind, randomized, placebo-controlled trial. | Multivitamin supplementation |
| **24** | Zajac et al. The Effects of Vitamin D-Enriched Mushrooms and Vitamin D3 on Cognitive Performance and Mood in Healthy Elderly Adults: A Randomised, Double-Blinded, Placebo-Controlled Trial. | Not sufficient information |

**Supplementary Table 4.** Characteristics of the trials included in the meta-analysis of vitamin D3 supplementation and depression.

| **Author, years, country** | **Participants (n)** | **Female (%)** | **Age (year)** | **Follow-up, weeks** | **Diagnosis of depression or anxiety** | **Antidepressant use** | **Baseline depression risk** | **Vitamin D3 deficiency** | **Intervention** | **Baseline BMI, kg/m^2^** | **Drop out** |
| --- | --- | --- | --- | --- | --- | --- | --- | --- | --- | --- | --- |
| Alghamdi, 2019,  Saudi Arabia (Alghamdi et al., 2020) | Adults diagnosed with MDD (n=62) | NR | 18–65 (41.5 ± 1.8) | 12 | BDI | Yes, SSRIs | High risk | Mixed | 50,000 IU/wk (Oral calciferol + SOC) | 32.6 ± 2.9 | NR |
| Bertone-Johnson, 2011,  US (Bertone-Johnson et al., 2012) | Postmenopausal women with depression  (n=2,263) | 100% | 50–79  (47) | 52-156 | Burnam Scale | Yes, (selective  serotonin reuptake inhibitors, monoamine oxidase inhibitors,  modified cyclic agents, tricyclic agent, and etc.) | High risk | No | 400 IU/day vitamin D3 + 1,000 mg/day elemental  calcium | NR | NR |
| Choukri, 2018,  New Zealand (Choukri, Conner, Haszard, Harper, & Houghton, 2018) | Healthy women (n=152) | 100% | 18–40 (24.2 ± 6.0) | 6 | CES-D,  , HADS anxiety subscale | No | Low risk | No | 50,000 IU/mo oral vitamin D3 tablets | I: 25.1 ± 4.5  C: 24.8 ± 4.9 | I: 0  C: 2 |
| Dean, 2011, Australia and New Zealand (Dean et al., 2011) | Healthy volunteers (n=128) | 57% | 18–30 (21.8 ± 2.9) | 6 | BDI, STAI | No | Low risk | No | 5,000 IU/day Oral cholecalciferol | NR | 1 |
| Eid, 2019, Saudi Arabia (Eid et al., 2019) | Patients diagnosed with GAD (n=30) | 43% | (40.1 ± 2.0) | 12 | GAD-7 | Yes,  SOC treatment  for GAD including antidepressant and anxiolytic medications | High risk | Yes | 50,000 IU/wk (Oral calciferol+ SOC) | NR | 0 |
| Fazelian, 2019,  Iran (Fazelian, Amani, Paknahad, Kheiri, & Khajehali, 2019) | Diabetic women with anxiety (n=51) | 100% | 20-60  (47.4 ± 9.5) | 16 | DASS*‑*21 | NR | High risk | Yes | Oral pearl of 50,000 IU vitamin  D3 fortnightly | I: 30.2 ± 4.42  C: 29.2 ± 6.4 | I: 7  C: 6 |
| Frandsen, 2014, Denmark (Frandsen, Pareek, Hansen, & Nielsen, 2014) | Healthcare  professionals with seasonal affective symptoms (n=43) | 94% | 18-65  (44.3 ± 10.6) | 12 | SPAQ-SAD | No | High risk | No | 70 μg/day vitamin D | NR | I: 6  C: 3 |
| Gaughran, 2021,  UK (Gaughran et al., 2021) | Adults diagnosed with early  psychosis (n=149) | 40.3% | 18-65, (28.1 ± 8.5) | 24 | CDS | NR | High risk | Mixed | 120,000 IU/mo of cholecalciferol | I: 25.9 ± 4.6  C: 26.4 ± 5.9 | C: 18  I: 17 |
| Ghaderi, 2019,  Iran (Ghaderi et al., 2020) | Patients under MMT (n=64) | 0% | 18-60 | 24 | BAI, BDI | Yes, Methadone was consumed in the form of  syrup by patient | High risk | Yes | 50,000 IU vitamin  D fortnightly | I: 24.9 ±4.0  C: 25.8 ± 3.5 | I: 3  C: 3 |
| Ghaderi, 2017,  Iran (Ghaderi et al., 2017) | Patients under MMT (n=68) | NR | 25–70  (41.3 ± 12.8) | 12 | BDI, BAI | Yes, Methadone was consumed in the form of  syrup by patients | High risk | Yes | 50,000 IU vitamin D fortnightly | I: 24.6 ± 4.4  C: 25.5 ± 4.4 | I: 4  C: 4 |
| Hansen, 2019, Denmark (Hansen et al., 2019) | Patients with depression (n=62) | 69.1% | 18–65  (39.1 ± 12.3) | 12 | HAM-D17 | Yes, psychotropic medication according to national guidelines | High risk | Mixed | 2800 IU vitamin D3/day | NR | I: 8  C: 9 |
| Jorde, 2018, Norway (Jorde & Kubiak, 2018) | Males and females 21 to 70 years old (n=408) | 46.8% | (52.0 ± 8.8) | 16 | BDI-II | Mixed, twenty-three subjects were using antidepressant  or mood-stabilizing drugs | Medium risk | No | 20, 000 IU/wk | C: 27.8 ± 4.8  I: 28.0 ± 4.8 | NR |
| Jorde, 2008, Norway (Jorde, Sneve, Figenschau, Svartberg, & Waterloo, 2008) | Overweight and obese subjects with depressive symptoms (n=441) | 63.9% | 21–70 (47.0) | 52 | BDI | No | High risk | Mixed | Two  capsules/wk,  each capsule:  (20,000 IU vitamin D) | I: 34.1  C: 34.7 | NR |
| Kusmiyati, 2020, Indonesia (Kusmiyati, Suryani, Herawati, & Firdausi, 2020) | Midwifery students who did  not suffer from any chronic disease, nor experience academic stress (n=77) | 100% | >18 | 4 | DASS-42 | No | Low risk | NR | 1 tablet (400 IU/day) vitamin D | NR | 0 |
| Kaviani, 2019,  Iran (Kaviani, Nikooyeh, Zand, Yaghmaei, & Neyestani, 2020) | Subjects diagnosed with  depression (n=56) | 89.2 % | 18-60  (43.0 ± 1.1) | 8 | BDI-II | No | High risk | Mixed | 50,000 IU cholecalciferol/2wks | I: 29.9 ± 4.6  C: 28.5 ± 5.3 | I: 8  C: 5 |
| Koning, 2019, Netherlands (de Koning et al., 2019) | High-risk older  participants with low vitamin D status (n=155) | 57.4 % | 60–80 | 52 | BAI, CES-D | No | Medium risk | Yes | 1200 IU/d vitamin D3  tablets | I: 27.1  C: 26.9 | I: 3  C: 7 |
| Krivoy, 2017,  Israel (Krivoy et al., 2017) | Schizophrenia  patients who had been maintained on clozapine treatment for at least 18 weeks (n=47) | 31.9 % | 18-65  (40.9 ± 14.7) | 8 | PANSS | Yes, maintained on clozapine treatment for at least 18 weeks | High risk | Yes | 14,000 IU/wk oral drops of vitamin D | I: 28.3 ± 3.8  C: 28.1 ± 6.0 | I: 2  C: 3 |
| Masoudi Alavi, 2018, Iran (Alavi, Khademalhoseini, Vakili, & Assarian, 2019) | Older adults  with depression (n=78) | 50% | >60  (67.8 ± 9.4) | 8 | GDS-15 | No | High risk | Yes | 50,000 IU/wk vitamin D3 pearl | NR | I: 1  C: 1 |
| Marsh, 2017, USA (Marsh, Penny, & Rothschild, 2017) | Patients with bipolar disorder spectrum diagnosis  (bipolar I, II, NOS) and were currently experiencing depressive symptoms rating 7 (mild) or  greater on the MADRS (n=31) | 51.6% | 18-70  (44.2 ± 18.5) | 12 | MADRS | Mixed, eight (50%) of the vitamin D group and six (35%) of the placebo group were taking  antidepressant medication | High risk | Yes | 5000IU Vitamin D3 capsule daily | NR | C: 5  I: 3 |
| Okereke, 2020, United States (Okereke et al., 2020) | Healthy men aged ≥50 years and women Aged ≥55 years (n=18,353) | 49.2% | (67.5 ± 7.1) | 256.8 | PHQ-8 | No | Low risk | Mixed | Vitamin D3 (2000 IU/d of cholecalciferol) | NR | I: 3746  C: 3772 |
| Omidian, 2019,  Iran (Omidian et al., 2019) | Subjects  with T2DM and depressive symptoms (n=68) | 40.9% | 30-60  (50.5 ±  8.7) | 12 | BDI II Persian | No | High risk | Yes | 4000 IU/d vitamin D | I: 27.3 ± 2.3  C:27.5 ± 1.6 | 2 |
| Rolf, 2017, Netherlands (Rolf et al., 2017) | Relapsing Remitting  MS patients (n=40) | 65% | 18-55  (38.0 ± 12.4) | 48 | HADS-D | No | Low risk | No | 14,000 IU/day  vitamin D3 | NR | I:3  C:2 |
| Sikaroudi, 2020,  Iran (Khalighi Sikaroudi et al., 2020) | IBS-D patients (n=74) | 52.7 % | 18-65 | 9 | HADS | No | Medium risk | Yes | 50,000 IU/week vitamin D3 | I: 24.5 ± 4.6  C: 26.2 ± 4.4 | C: 9  I: 5 |
| Sharifi, 2018,  Iran (Sharifi, Vahedi, Nedjat, Mohamadkhani, & Hosseinzadeh Attar, 2019) | Mild to moderate ulcerative colitis patients (n=90) | 43.3 % | (36.2 ± 12.8) | 12 | BDI | NR | Medium risk | Mixed | Single injection of 300,000 IU vitamin D3 | I: 25.0 ± 3.5  C: 25.6 ± 3.5 | I: 0  C: 4 |
| Sepehrmanesh, 2015,  Iran (Sepehrmanesh et al., 2016) | Patients with a diagnosis of MDD (n=40) | 85 % | 18-65  (36.3 ± 11.1) | 8 | BDI | NR | High risk | Yes | Single capsule of 50 000IU vitamin D/wk | I: 25.8 ± 6.5  C: 26.9 ± 6.3 | I:2  C: 2 |
| Vellekkatt, 2020,  India (Vellekkatt, Menon, Rajappa, & Sahoo, 2020) | Patients with MDD and concurrent vitamin D deficiency (n=46) | 67.3 % | 18-65 | 12 | HRDS-17 | Yes, patients were initiated on anti-depressants and other elements of standard care (including psychotherapy) | High risk | Yes | TAU + single parenteral dose of 300,000 IU vitamin D | I: 21.9 ± 4.2  C: 23.8 ± 5.6 | I: 1  C: 3 |
| Vieth, 2004, Canada (Vieth, Kimball, Hu, & Walfish, 2004) | Thyroid clinic outpatients  (n=64) | 83% | 53.0 ± 16.6 | 52 | Well-being questionnaire | No | Medium risk | No | 100 mcg/day vitamin D |  | NR |
| Wang, 2016, china (Wang et al., 2016) | Dialysis patients with depression treated and Vitamin D3 Insufficiency (n=726) | I: 39%  C: 42.3 % | ≥18 | 52 | BDI II | No | High risk | Yes | 50,000 IU/wk  cholecalciferol | I: 23.7 ± 10.6  C: 24.2 ± 9.8 | NR |
| Zhu, 2020, China (Zhu et al., 2020) | Participants with low 25(OH)D levels (n=158) | 73.5 % | 18–60  (44.8 ± 16.9) | 24 | HAMD-17  HAMA-14 | NR | High risk | Yes,  serum 25(OH) D levels ≤75 nmol/L | 1,600 IU/d vitamin D | I: 24.1 ±4 .2  C: 23.6 ± 4.1 | I: 17  C: 35 |
| Zheng, 2018, Australia (Zheng et al., 2019) | Patients with knee osteoarthritis (n = 413) | 50.3% | (63.2 ± 7.0) | 144 | PHQ-9 | Mixed | High risk | Yes | Vitamin D3 50,000 IU/mo | I: 29.6 ± 5.4  C: 29.6 ± 4.6 | I: 28  C: 45 |
| Zhang, 2018, china (Zhang, Wang, Zhu, & Yang, 2018) | Pulmonary tuberculosis patients with depression (n=120) | 16.2 % | ≥18  (32.2 ± 13.3) | 8 | Chinese version of BDI | NR | High risk | Yes | 100,000 IU/wk cholecalciferol, orally | I: 21.2 ± 3.8  C: 20.7 ± 4.1 | 2 |
| **Abbreviations:** BAI, Beck Anxiety Inventory; BDI, The Beck Depression Inventory; C, Control; CABG, coronary artery bypass graft surgery; CDS, Calgary Depression Scale; CES-D, Center for Epidemiologic Studies Depression Scale; CMAI, Cohen-Mansfield Agitation Inventory; DASS, Depression, Anxiety and Stress Scales; DBP, Diastolic Blood Pressure; GAD, Generalized Anxiety Disorder; GAD-7, the Generalized Anxiety Disorder 7-item; GDS, Geriatric Depression Scale; HADs, PHQ-8, Patient Health Questionnaire; hospital anxiety and depression Scale; HADS, Hospital Anxiety Depression Scale; HADS, The Hospital Anxiety and Depression Scale; HADS-D, Hospital Anxiety Depression subscale; HAM-A, Hamilton Anxiety Rating Scale; HAMA-14, Hamilton Anxiety Rating Scale-14; HAM-D, Hamilton depression score; HRDS-17, Hamilton Depression rating scale-17. HRQOL, health-related quality of life; I, Intervention; IBS-D, diarrhea-predominant irritable bowel syndrome; MADRS, Montgomery Aasberg Depression Rating Scale; MDD, Major Depressive Disorder; MMSE, Mini-Mental State Examination; MMT, maintenance methadone treatment; MS, multiple sclerosis; PANSS, Positive and Negative Syndrome Scale; PDI-21, The Peters Delusion Inventory-21; PGI-I, Patient Global Impression Improvement scale; PHQ, Patient Health Questionnaire; PSQI, Pittsburgh Sleep Quality Index; SBP, systolic Blood Pressure; SF-12, 12-item Short Form Health Survey; SOC, standard of care; SPAQ-SAD, the Seasonal Pattern Assessment Questionnaire; SPPB, Short Physical Performance Battery; STAI, The State-Trait Anxiety Inventory; T2DM, Type 2 diabetes mellitus; TAU, Treatment As Usual; UMACL, UWIST Mood Adjective Check List. | | | | | | | | | | | |

**Supplementary Table 5.** Risk of bias assessment of trials included in the meta-analysis of the effect of vitamin D_3_ supplementation on depression.

| **Study, year** | **Bias arising from the randomization process** | **Bias due to deviations from intended intervention** | **Bias due to missing outcome data** | **Bias in measurement of the outcome** | **Bias in selection of the reported result** | **Overall quality** |
| --- | --- | --- | --- | --- | --- | --- |
| Dean, 2011 | Low | Low | Low | Low | Low | Low risk of bias |
| Frandsen, 2014 | Low | Low | Low | Low | Low | Low risk of bias |
| Gaughran, 2021 | Low | Low | Low | Low | Low | Low risk of bias |
| Hansen, 2019 | Low | Low | Low | Low | Low | Low risk of bias |
| Vellekkatt, 2020 | Low | Low | Low | Low | Low | Low risk of bias |
| Choukri, 2018 | Some concerns | Low | Low | Some concerns | Low | Some concerns |
| Fazelian, 2019 | Some concerns | Some concerns | Low | Low | Low | Some concerns |
| Ghaderi, 2019 | Low | Some concerns | Low | Low | Low | Some concerns |
| Ghaderi, 2017 | Some concerns | Low | Low | Low | Low | Some concerns |
| Jorde, 2008 | Some concerns | Low | Low | Low | Low | Some concerns |
| Koning, 2019 | Some concerns | Low | Low | Low | Low | Some concerns |
| Krivoy, 2017 | Some concerns | Low | Low | Low | Low | Some concerns |
| Omidian, 2019 | Some concerns | Some concerns | Low | Low | Low | Some concerns |
| Okereke, 2020 | Low | Some concerns | Low | Some concerns | Low | Some concerns |
| Vieth, 2004 | Some concerns | Low | Low | Low | Low | Some concerns |
| Wang, 2016 | Some concerns | Some concerns | Low | Low | Low | Some concerns |
| Zheng, 2018 | Some concerns | Some concerns | Low | Low | Low | Some concerns |
| Jorde, 2018 | High | High | High | Low | Low | High risk of bias |
| Alghamdi, 2019 | Some concerns | High | High | Some concerns | Low | High risk of bias |
| Bertone-Johnson, 2011 | High | Some concerns | Low | Some concerns | Low | High risk of bias |
| Eid, 2019 | Some concerns | High | High | High | Low | High risk of bias |
| Kusmiyati, 2020 | Some concerns | High | Low | Low | Low | High risk of bias |
| Kaviani, 2019 | Some concerns | High | Low | Low | Low | High risk of bias |
| Marsh, 2017 | Some concerns | High | Low | Low | Low | High risk of bias |
| Masoudi Alavi, 2018 | Some concerns | High | Low | Low | Low | High risk of bias |
| Rolf, 2017, | Some concerns | High | High | Low | Low | High risk of bias |
| Sikaroudi, 2020 | Some concerns | High | Some concerns | Low | Low | High risk of bias |
| Sharifi, 2018 | Some concerns | High | Low | Low | Low | High risk of bias |
| Sepehrmanesh, 2015 | Some concerns | High | Low | Low | Low | High risk of bias |
| Zhu, 2020 | High | High | High | Low | Low | High risk of bias |
| Zheng, 2018 | Low | High | High | Low | Low | High risk of bias |

**Supplementary Table 6**. Assessment of credibility of subgroup difference for the effects of vitamin D_3_ supplementation on depressive symptoms based on ICEMAN.

| Variable | Q1 | Q2 | Q3 | Q4 | Q5 | Q6 | Q7 | Q8 | Overall credibility |
| --- | --- | --- | --- | --- | --- | --- | --- | --- | --- |
| Intervention duration | Completely between | Probably not similar | Rather small | Definitely yes | Chance an unlikely explanation | Definitely yes | Definitely yes | Definitely no | Low |
| Baseline risk of depression | Completely between | Probably not similar | Rather large | Definitely yes | Chance a likely explanation | Definitely yes | Definitely yes | not applicable | Low |
| Sex | Completely between | Probably not similar | Very small | Definitely no | Chance an unlikely explanation | Definitely no | Definitely yes | not applicable | Low |
| Weight status | Completely between | Probably not similar | Rather large | Definitely no | Chance a likely explanation | Definitely no | Definitely yes | not applicable | Low |
| Depression status | Completely between | Probably not similar | Very small | Definitely no | Chance an unlikely explanation | Definitely no | Definitely yes | not applicable | Low |
| Vitamin D deficiency | Completely between | Probably not similar | Rather large | Definitely no | Chance an unlikely explanation | Definitely no | Definitely yes | not applicable | Low |

Q, question; Q1, Is the analysis of effect modification based on comparison within rather than between trials? Q2, For within-trial comparisons, is the effect modification similar from trial to trial? Q3, For between-trial comparisons, is the number of trials large? Q4, Was the direction of the effect modification correctly hypothesized priori? Q5, Does a test for interaction suggest that chance is an unlikely explanation of the apparent effect modification? Q6, Did the authors test only a small number of effect modifiers? Q7, Did the authors use a random effects model? Q8, If the effect modifier is a continuous variable, were arbitrary cut points avoided?

**Supplementary Table 7**. GRADE evidence table for the effects of vitamin D supplementation on primary and secondary outcomes.

| Certainty assessment | | | | | | | № of patients | | Effect | | **Certainty** |
| --- | --- | --- | --- | --- | --- | --- | --- | --- | --- | --- | --- |
| № of studies | Study design | Risk of bias | Inconsistency | Indirectness | Imprecision | Other considerations | [intervention] | [comparison] | Relative (95% CI) | Absolute (95% CI) |  |
| **Depressive symptoms** | | | | | | | | | | | |
| 31 | randomised trials | not serious^a^ | serious^b^ | not serious | serious^c^ | dose response gradient | 12,098 | 12,091 | - | SMD 0.32 SD lower (0.43 lower to 0.22 lower) | ⨁⨁⨁◯ Moderate |
| **Depression remission** | | | | | | | | | | | |
| 1 | randomised trials | serious^d^ | not serious | not serious | serious^e^ | none | 10/20 (50.0%) | 2/10 (20.0%) | **OR 0.20** (0.03 to 1.15) | **33 fewer per 100** (from 64 fewer to 03 fewer) | ⨁⨁◯◯ Low |
| **Severity of anxiety** | | | | | | | | | | | |
| 7 | randomised trials | not serious | serious^f^ | not serious | serious^e^ | none | 332 | 309 | - | SMD 0.33 SD lower (0.92 lower to 0.25 higher) | ⨁⨁◯◯ Low |
| **Adverse events** | | | | | | | | | | | |
| 8 | randomised trials | not serious | not serious | not serious | very serious^g^ | none | 346/1829 (18.9%) | 302/1843 (16.4%) | **OR 1.20** (1.00 to 1.45) | **1 more per 100** (from 1 more to 2 more) | ⨁⨁◯◯ Low |
| **Serious adverse events** | | | | | | | | | | | |
| 2 | randomised trials | serious^h^ | serious^i^ | not serious | very serious^g^ | none | 252/1051 (24.0%) | 220/1064 (20.7%) | **OR 1.00** (0.51 to 1.97) | **0 fewer per 100** (from 11 fewer to 11 more) | ⨁◯◯◯ Very low |
| **Comorbidity index** | | | | | | | | | | | |
| 1 | randomised trials | serious^h^ | not serious | not serious | serious^e^ | none | 362 | 364 | - | SMD 0.18 SD higher (0.9 lower to 1.26 higher) | ⨁⨁◯◯ Low |
| **Stress** | | | | | | | | | | | |
| 1 | randomised trials | serious^h^ | not serious | not serious | serious^e^ | none | 26 | 25 | - | SMD 0.18 SD higher (0.9 lower to 1.26 higher) | ⨁⨁◯◯ Low |
| **Overall quality of life** | | | | | | | | | | | |
| 2 | randomised trials | serious^h^ | not serious | not serious | serious^e^ | none | 62 | 58 | - | SMD 0.24 SD lower (0.63 lower to 0.15 higher) | ⨁⨁◯◯ Low |
| **Bodily image** | | | | | | | | | | | |
| 1 | randomised trials | serious^h^ | not serious | not serious | serious^h^ | none | 39 | 35 | - | SMD 0.3 SD lower (0.99 lower to 0.39 higher) | ⨁⨁◯◯ Low |
| **Dysphoria** | | | | | | | | | | | |
| 1 | randomised trials | serious^h^ | not serious | not serious | serious^e^ | none | 39 | 35 | - | SMD 0.4 SD lower (1.09 lower to 0.29 higher) | ⨁⨁◯◯ Low |
| **Food avoidance** | | | | | | | | | | | |
| 1 | randomised trials | serious^h^ | not serious | not serious | serious^c^ | none | 39 | 35 | - | SMD 0.02 SD higher (0.41 lower to 0.45 higher) | ⨁⨁◯◯ Low |
| **Healthy worry** | | | | | | | | | | | |
| 1 | randomised trials | serious^h^ | not serious | not serious | serious^e^ | none | 39 | 35 | - | SMD 0.16 SD lower (0.63 lower to 0.31 higher) | ⨁⨁◯◯ Low |
| **Relationship** | | | | | | | | | | | |
| 1 | randomised trials | serious^h^ | not serious | not serious | serious^e^ | none | 39 | 35 | - | SMD 0.13 SD lower (0.5 lower to 0.24 higher) | ⨁⨁◯◯ Low |
| **Sexual** | | | | | | | | | | | |
| 1 | randomised trials | serious^h^ | not serious | not serious | serious^e^ | none | 39 | 35 | - | SMD 0 SD  (1.47 lower to 1.47 higher) | ⨁⨁◯◯ Low |
| **Social reaction** | | | | | | | | | | | |
| 1 | randomised trials | serious^h^ | not serious | not serious | serious^e^ | none | 39 | 35 | - | SMD 0 SD  (0.35 lower to 0.35 higher) | ⨁⨁◯◯ Low |

**CI:** confidence interval; **OR:** odds ratio; **SMD:** standardized mean difference.

#### Explanations:

a. Although most studies were rated to have some concerns, there is no statistical difference across subgroups defined by study quality. Not downgraded.

b. Serious inconsistency since I^2^=98%. Downgraded.

c. Serious imprecision since point estimate was smaller than the MCID threshold. Downgraded.

d. Serious risk of bias since there is only one trial which rated to have some concerns due to incomplete outcome data and blinding of outcome assessment. Downgraded.

e. Serious imprecision since optimal information size was not met. Downgraded.

f. Serious inconsistency since I^2^=87%. Downgraded.

g. Very serious imprecision since the upper bound of the 95%CI surpassed important harm. Downgraded to two levels.

h. Serious risk of bias due to selective outcome reporting. Downgraded.

i. Serious inconsistency since I^2^=55%. Downgraded.

a

Meta-analyses included in quantitative synthesis (meta-analysis)
(n=31)

Records excluded
(n=1582)

Records screened
(n=1637)

Records after duplicates removed
(n=1637)

Additional records identified through manual search
(n=1)

Records identified through database searching
(n=2243)

## **Identification**

## **Screening**

Full-text articles assessed for eligibility
(n=55)

## **Eligibility**

Full-text articles excluded, with reasons (n=24)

Duplicate (n=4)

Not RCT (n=2)

Under 4 weeks (n=1)

Multivitamin supplementation (n=4)

Not sufficient information (n=10)

Not interested intervention (n=2)

Not interested outcome (n=1)

Not interested outcome (n=1)

Less than two weeks (n=15)

Different population (n=7)

Meta-analyses included in qualitative synthesis
(n=55)

## **Included**

**Supplementary Figure 1.** Literature search and study selection process.


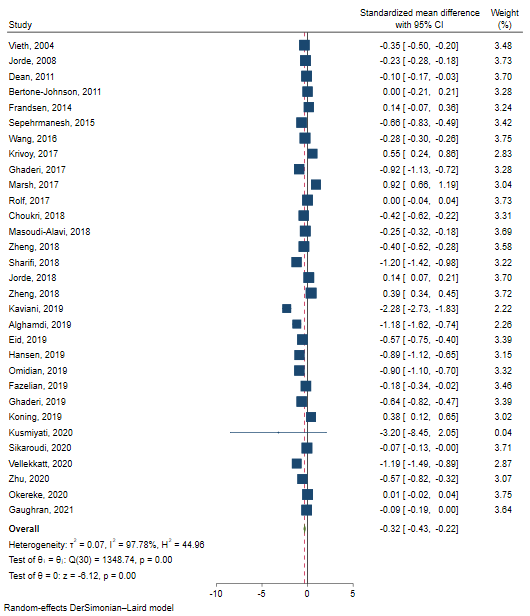


**Supplementary Figure 2.** Standardized mean difference on the effects of vitamin D3 (each 1000 IU/d) on depressive symptoms using random effects model. CI: confidence interval.


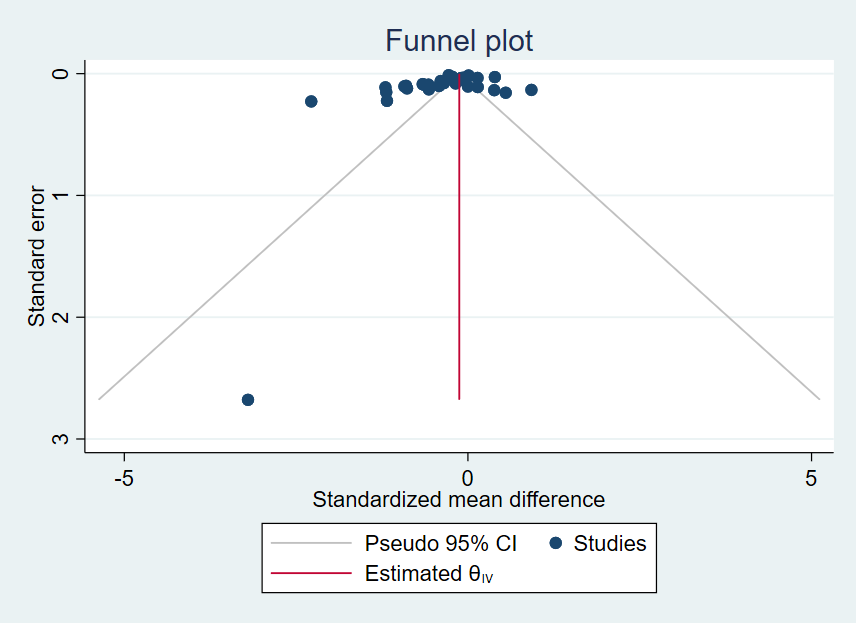


**Supplementary Figure 3.** Funnel plot of the effects of vitamin D3 (each 1000 IU/d) on depressive symptoms.

**Supplementary Figure S4.** The effects of vitamin D3 (each 1000 IU/d) on depression remission (data are presented as odds ratio with 95% confidence intervals).

**Supplementary Figure 5.** The effects of vitamin D3 (each 1000 IU/d) on depression remission (data are presented as risk difference with 95% confidence interval).

**Supplementary Figure 6.** The effects of vitamin D3 (each 1000 IU/d) on severity of anxiety.

**Supplementary Figure 7.** The effects of vitamin D3 (each 1000 IU/d) on adverse event (data are presented as odds ratio with 95% confidence interval).

**Supplementary Figure 8.** The effects of vitamin D3 (each 1000 IU/d) on adverse event (data are presented as risk difference with 95% confidence interval).

**Supplementary Figure 9.** The effects of vitamin D3 (each 1000 IU/d) on serious adverse event (data are presented as odds ratio with 95% confidence interval).

**Supplementary Figure 10.** The effects of vitamin D3 (each 1000 IU/d) on serious adverse event (data are presented as risk difference with 95% confidence interval).

**Supplementary Figure 11.** The effects of vitamin D3 (each 1000 IU/d) on comorbidity index.

**Supplementary Figure 12.** The effects of vitamin D3 (each 1000 IU/d) on stress.

**Supplementary Figure 13.** The effects of vitamin D3 (each 1000 IU/d) on quality of life.

**Supplementary Figure 14.** The effects of vitamin D3 (each 1000 IU/d) on bodily image.

**Supplementary Figure 15.** The effects of vitamin D3 (each 1000 IU/d) on dysphoria.

**Supplementary Figure 16.** The effects of vitamin D3 (each 1000 IU/d) on food avoidance.

**Supplementary Figure 17.** The effects of vitamin D3 (each 1000 IU/d) on health worry.

**Supplementary Figure 18.** The effects of vitamin D3 (each 1000 IU/d) on relationship.

**Supplementary Figure 19.** The effects of vitamin D3 (each 1000 IU/d) on sexual.

**Supplementary Figure 20.** The effects of vitamin D3 (each 1000 IU/d) on social reaction.

**Supplementary References:**

Alavi, N. M., Khademalhoseini, S., Vakili, Z., & Assarian, F. (2019). Effect of vitamin D supplementation on depression in elderly patients: A randomized clinical trial. *Clinical Nutrition, 38*(5), 2065-2070. doi:10.1016/j.clnu.2018.09.011

Alghamdi, S., Alsulami, N., Khoja, S., Alsufiani, H., Tayeb, H. O., & Tarazi, F. I. (2020). Vitamin D Supplementation Ameliorates Severity of Major Depressive Disorder. *Journal of Molecular Neuroscience, 70*(2), 230-235. doi:10.1007/s12031-019-01461-2

Bertone-Johnson, E. R., Powers, S. I., Spangler, L., Larson, J., Michael, Y. L., Millen, A. E., . . . Manson, J. E. (2012). Vitamin D supplementation and depression in the women's health initiative calcium and vitamin D trial. *American Journal of Epidemiology, 176*(1), 1-13. doi:10.1093/aje/kwr482

Choukri, M. A., Conner, T. S., Haszard, J. J., Harper, M. J., & Houghton, L. A. (2018). Effect of vitamin D supplementation on depressive symptoms and psychological wellbeing in healthy adult women: a double-blind randomised controlled clinical trial. *Journal of Nutritional Science, 7*, e23. doi:10.1017/jns.2018.14

de Koning, E. J., Lips, P., Penninx, B., Elders, P. J. M., Heijboer, A. C., den Heijer, M., . . . van Schoor, N. M. (2019). Vitamin D supplementation for the prevention of depression and poor physical function in older persons: the D-Vitaal study, a randomized clinical trial. *American Journal of Clinical Nutrition, 110*(5), 1119-1130. doi:10.1093/ajcn/nqz141

Dean, A. J., Bellgrove, M. A., Hall, T., Phan, W. M., Eyles, D. W., Kvaskoff, D., & McGrath, J. J. (2011). Effects of vitamin D supplementation on cognitive and emotional functioning in young adults--a randomised controlled trial. *PLoS One, 6*(11), e25966. doi:10.1371/journal.pone.0025966

Eid, A., Khoja, S., AlGhamdi, S., Alsufiani, H., Alzeben, F., Alhejaili, N., . . . Tarazi, F. I. (2019). Vitamin D supplementation ameliorates severity of generalized anxiety disorder (GAD). *Metabolic Brain Disease, 34*(6), 1781-1786. doi:10.1007/s11011-019-00486-1

Fazelian, S., Amani, R., Paknahad, Z., Kheiri, S., & Khajehali, L. (2019). Effect of Vitamin D Supplement on Mood Status and Inflammation in Vitamin D Deficient Type 2 Diabetic Women with Anxiety: A Randomized Clinical Trial. *International Journal of Preventive Medicine, 10*, 17. doi:10.4103/ijpvm.IJPVM_174_18

Frandsen, T. B., Pareek, M., Hansen, J. P., & Nielsen, C. T. (2014). Vitamin D supplementation for treatment of seasonal affective symptoms in healthcare professionals: a double-blind randomised placebo-controlled trial. *BMC Research Notes, 7*, 528. doi:10.1186/1756-0500-7-528

Gaughran, F., Stringer, D., Wojewodka, G., Landau, S., Smith, S., Gardner-Sood, P., . . . McGrath, J. (2021). Effect of Vitamin D Supplementation on Outcomes in People With Early Psychosis: The DFEND Randomized Clinical Trial. *JAMA Network Open, 4*(12), e2140858. doi:10.1001/jamanetworkopen.2021.40858

Ghaderi, A., Banafshe, H. R., Motmaen, M., Rasouli-Azad, M., Bahmani, F., & Asemi, Z. (2017). Clinical trial of the effects of vitamin D supplementation on psychological symptoms and metabolic profiles in maintenance methadone treatment patients. *Progress in Neuro-Psychopharmacology & Biological Psychiatry, 79*(Pt B), 84-89. doi:10.1016/j.pnpbp.2017.06.016

Ghaderi, A., Rasouli-Azad, M., Farhadi, M. H., Mirhosseini, N., Motmaen, M., Pishyareh, E., . . . Asemi, Z. (2020). Exploring the Effects of Vitamin D Supplementation on Cognitive Functions and Mental Health Status in Subjects Under Methadone Maintenance Treatment. *Journal of Addiction Medicine, 14*(1), 18-25. doi:10.1097/adm.0000000000000550

Guyatt, G. H., Oxman, A. D., Kunz, R., Brozek, J., Alonso-Coello, P., Rind, D., . . . Vist, G. (2011). GRADE guidelines 6. Rating the quality of evidence—imprecision. *Journal of clinical epidemiology, 64*(12), 1283-1293.

Guyatt, G. H., Oxman, A. D., Kunz, R., Woodcock, J., Brozek, J., Helfand, M., . . . Vist, G. (2011). GRADE guidelines: 8. Rating the quality of evidence—indirectness. *Journal of clinical epidemiology, 64*(12), 1303-1310.

Guyatt, G. H., Oxman, A. D., Kunz, R., Woodcock, J., Brozek, J., Helfand, M., . . . Akl, E. A. (2011). GRADE guidelines: 7. Rating the quality of evidence—inconsistency. *Journal of clinical epidemiology, 64*(12), 1294-1302.

Guyatt, G. H., Oxman, A. D., Montori, V., Vist, G., Kunz, R., Brozek, J., . . . Falck-Ytter, Y. (2011). GRADE guidelines: 5. Rating the quality of evidence—publication bias. *Journal of clinical epidemiology, 64*(12), 1277-1282.

Guyatt, G. H., Oxman, A. D., Vist, G., Kunz, R., Brozek, J., Alonso-Coello, P., . . . Falck-Ytter, Y. (2011). GRADE guidelines: 4. Rating the quality of evidence—study limitations (risk of bias). *Journal of clinical epidemiology, 64*(4), 407-415.

Guyatt, G. H., Oxman, A. D., Vist, G. E., Kunz, R., Falck-Ytter, Y., Alonso-Coello, P., & Schünemann, H. J. (2008). GRADE: an emerging consensus on rating quality of evidence and strength of recommendations. *Bmj, 336*(7650), 924-926. doi:10.1136/bmj.39489.470347.AD

Hansen, J. P., Pareek, M., Hvolby, A., Schmedes, A., Toft, T., Dahl, E., & Nielsen, C. T. (2019). Vitamin D3 supplementation and treatment outcomes in patients with depression (D3-vit-dep). *BMC Research Notes, 12*(1), 203. doi:10.1186/s13104-019-4218-z

Jorde, R., & Kubiak, J. (2018). No improvement in depressive symptoms by vitamin D supplementation: results from a randomised controlled trial. *Journal of Nutritional Science, 7*, e30. doi:10.1017/jns.2018.19

Jorde, R., Sneve, M., Figenschau, Y., Svartberg, J., & Waterloo, K. (2008). Effects of vitamin D supplementation on symptoms of depression in overweight and obese subjects: randomized double blind trial. *Journal of Internal Medicine, 264*(6), 599-609. doi:10.1111/j.1365-2796.2008.02008.x

Kaviani, M., Nikooyeh, B., Zand, H., Yaghmaei, P., & Neyestani, T. R. (2020). Effects of vitamin D supplementation on depression and some involved neurotransmitters. *Journal of Affective Disorders, 269*, 28-35. doi:10.1016/j.jad.2020.03.029

Khalighi Sikaroudi, M., Mokhtare, M., Shidfar, F., Janani, L., Faghihi Kashani, A., Masoodi, M., . . . Shidfar, S. (2020). Effects of vitamin D3 supplementation on clinical symptoms, quality of life, serum serotonin (5-hydroxytryptamine), 5-hydroxy-indole acetic acid, and ratio of 5-HIAA/5-HT in patients with diarrhea-predominant irritable bowel syndrome: A randomized clinical trial. *EXCLI Journal, 19*, 652-667. doi:10.17179/excli2020-2247

Krivoy, A., Onn, R., Vilner, Y., Hochman, E., Weizman, S., Paz, A., . . . Weizman, A. (2017). Vitamin D Supplementation in Chronic Schizophrenia Patients Treated with Clozapine: A Randomized, Double-Blind, Placebo-controlled Clinical Trial. *EBioMedicine, 26*, 138-145. doi:10.1016/j.ebiom.2017.11.027

Kusmiyati, Y., Suryani, E., Herawati, L., & Firdausi, A. (2020). Vitamin D and Reduced Academic Stress of Health Students. *Kesmas: National Public Health Journal, 15*. doi:10.21109/kesmas.v15i3.3274

Marsh, W. K., Penny, J. L., & Rothschild, A. J. (2017). Vitamin D supplementation in bipolar depression: A double blind placebo controlled trial. *Journal of Psychiatric Research, 95*, 48-53. doi:10.1016/j.jpsychires.2017.07.021

Okereke, O. I., Reynolds, C. F., 3rd, Mischoulon, D., Chang, G., Vyas, C. M., Cook, N. R., . . . Manson, J. E. (2020). Effect of Long-term Vitamin D3 Supplementation vs Placebo on Risk of Depression or Clinically Relevant Depressive Symptoms and on Change in Mood Scores: A Randomized Clinical Trial. *JAMA, 324*(5), 471-480. doi:10.1001/jama.2020.10224

Omidian, M., Mahmoudi, M., Abshirini, M., Eshraghian, M. R., Javanbakht, M. H., Zarei, M., . . . Djalali, M. (2019). Effects of vitamin D supplementation on depressive symptoms in type 2 diabetes mellitus patients: Randomized placebo-controlled double-blind clinical trial. *Diabetes & Metabolic Syndrome: Clinical Research & Reviews, 13*(4), 2375-2380. doi:10.1016/j.dsx.2019.06.011

Rolf, L., Muris, A. H., Bol, Y., Damoiseaux, J., Smolders, J., & Hupperts, R. (2017). Vitamin D(3) supplementation in multiple sclerosis: Symptoms and biomarkers of depression. *Journal of the Neurological Sciences, 378*, 30-35. doi:10.1016/j.jns.2017.04.017

Sepehrmanesh, Z., Kolahdooz, F., Abedi, F., Mazroii, N., Assarian, A., Asemi, Z., & Esmaillzadeh, A. (2016). Vitamin D Supplementation Affects the Beck Depression Inventory, Insulin Resistance, and Biomarkers of Oxidative Stress in Patients with Major Depressive Disorder: A Randomized, Controlled Clinical Trial. *The Journal of Nutrition, 146*(2), 243-248. doi:10.3945/jn.115.218883

Sharifi, A., Vahedi, H., Nedjat, S., Mohamadkhani, A., & Hosseinzadeh Attar, M. J. (2019). Vitamin D Decreases Beck Depression Inventory Score in Patients with Mild to Moderate Ulcerative Colitis: A Double-Blind Randomized Placebo-Controlled Trial. *Journal of Dietary Supplements, 16*(5), 541-549. doi:10.1080/19390211.2018.1472168

Vellekkatt, F., Menon, V., Rajappa, M., & Sahoo, J. (2020). Effect of adjunctive single dose parenteral Vitamin D supplementation in major depressive disorder with concurrent vitamin D deficiency: A double-blind randomized placebo-controlled trial. *Journal of Psychiatric Research, 129*, 250-256. doi:10.1016/j.jpsychires.2020.07.037

Vieth, R., Kimball, S., Hu, A., & Walfish, P. G. (2004). Randomized comparison of the effects of the vitamin D3 adequate intake versus 100 mcg (4000 IU) per day on biochemical responses and the wellbeing of patients. *Nutr J, 3*, 8. doi:10.1186/1475-2891-3-8

Wang, Y., Liu, Y., Lian, Y., Li, N., Liu, H., & Li, G. (2016). Efficacy of High-Dose Supplementation With Oral Vitamin D3 on Depressive Symptoms in Dialysis Patients With Vitamin D3 Insufficiency: A Prospective, Randomized, Double-Blind Study. *Journal of Clinical Psychopharmacology, 36*(3), 229-235. doi:10.1097/jcp.0000000000000486

Zhang, L., Wang, S., Zhu, Y., & Yang, T. (2018). Vitamin D3 as adjunctive therapy in the treatment of depression in tuberculosis patients: a short-term pilot randomized double-blind controlled study. *Neuropsychiatric Disease and Treatment, 14*, 3103-3109. doi:10.2147/ndt.S183039

Zheng, S., Tu, L., Cicuttini, F., Han, W., Zhu, Z., Antony, B., . . . Ding, C. (2019). Effect of Vitamin D Supplementation on Depressive Symptoms in Patients With Knee Osteoarthritis. *Journal of the American Medical Directors Association, 20*(12), 1634-1640.e1631. doi:10.1016/j.jamda.2018.09.006

Zhu, C., Zhang, Y., Wang, T., Lin, Y., Yu, J., Xia, Q., . . . Zhu, D. M. (2020). Vitamin D supplementation improves anxiety but not depression symptoms in patients with vitamin D deficiency. *Brain and Behavior, 10*(11), e01760. doi:10.1002/brb3.1760
